# Supplementary material for: Tunable band gaps and optical absorption properties of bent MoS2 nanoribbons
Source: Sci Rep. 2022 Feb 22;12:3008. doi: 10.1038/s41598-022-06741-3 (PMC8863845; doi:10.1038/s41598-022-06741-3)

SUPPORTING INFORMATION

Tunable band gaps and optical absorption properties of bent MoS_2_ nanoribbons

Hong Tang, Bimal Neupane, Santosh Neupane, Shiqi Ruan, Niraj K. Nepal, and Adrienn Ruzsinszky

Department of Physics, Temple University, Philadelphia, PA 19122


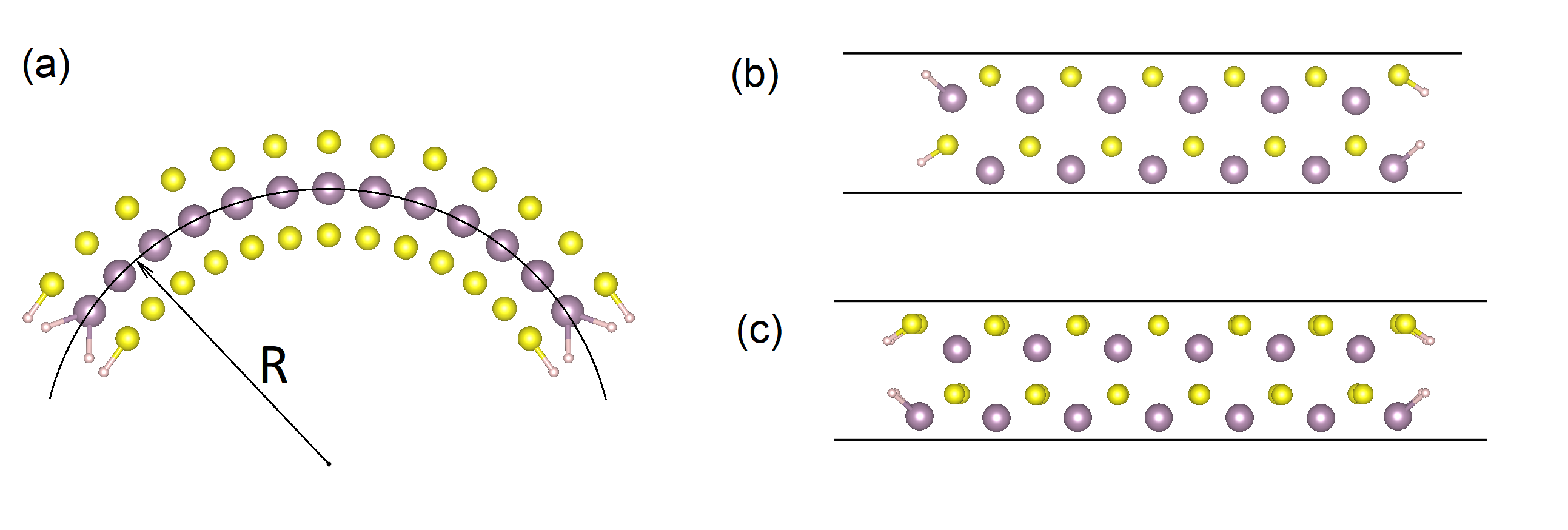


Figure S1. (a) The simplified structure of a bent nanoribbon. R is the bending curvature radius, which is measured from the center of the circle to the middle Mo atom layer. The outer S atom layer is the top layer and the inner S atom layer is the bottom layer, as shown. (b) A12MoS_2_ nanoribbon has two asymmetric edges. (c) A13MoS_2_ has two symmetric edges.

(a) (b)

Figure S2. The evolutions of NEG (a) and EG (b) for A*n*MoS_2_ nanoribbons with *n* from 12 to 24 with bending curvature 1/R.

(a) (b)

Figure S3. The evolutions of NEG (a) and EG (b) for A*n*MoS_2_ nanoribbons with *n* from 9 to 12 with bending curvatures, $\kappa=1/R$. For *n*=11, within the curvature range $0.1<\kappa<0.125/Å$, EG shows a sudden increase, this is due to the drastic geometry change of the A11MoS_2_ nanoribbon, especially around the two edges, as shown in Figure S9. With $\kappa>0.125/Å$, EG recovers to the normal trend and decreases with $\kappa$. However, with $\kappa>0.125/Å$ for *n*=9, and with $\kappa>0.1/Å$ for *n*=10, EG also shows a large increase. The structures of nanoribbons change so drastically with curvatures that the structures will undergo damaging deformations.

Figure S4. The length of vector c of the supercell (LC) as a function of curvatures for nanoribbons A*n*MoS_2_ for *n* from 12 to 24 is shown in (a) and for *n* from 9 to 12 in (b). With $\kappa>0.125/Å$ for *n*=9, and with $\kappa>0.1/Å$ for *n*=10, LC shows a large increase. The structures of nanoribbons change so drastically with curvatures that the structures will undergo damaging deformations. This is in line with the change of EG with curvatures (see Figure S3b).


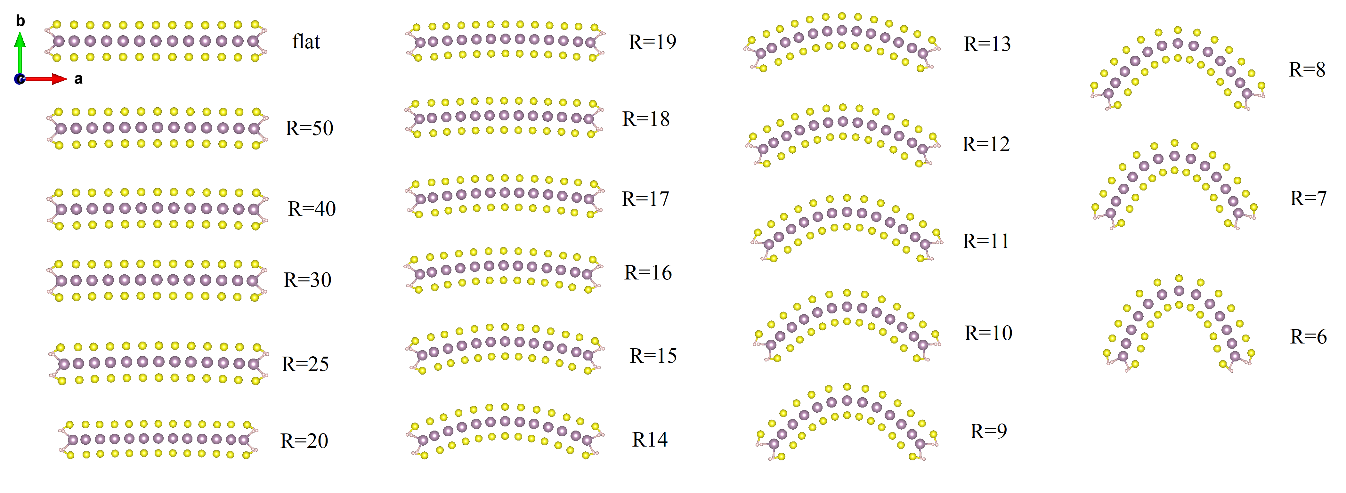


Figure S5. The relaxed structures of A13MoS_2_ nanoribbon for different curvature radii. R=50 stands for $R=50 Å$, and so on.

Figure S6. The evolution of strains with curvature radii for the A13MoS_2_ nanoribbon. The strain in the xy-plane for the outer S atom layer (SXYOS) with curvature radii is shown in (a) and the strain along the z direction for the outer S atom layer (SZOS) is shown in (b). The strain in the xy-plane for the inner S atom layer (SXYIS) is shown in (c) and the strain along the z direction for the inner S atom layer (SZIS) is in (d).

Figure S7. Similarly as Figure S6, the plots for A12MoS_2_ for the strains SXYM are shown in (a), SZM in (b), SXYOS in (c), SZOS in (b), SXYIS in (e) and SZIS in (f). The strains along the z direction (SZM, SZOS, and SZIS) have more complex patterns, due to the asymmetrical structures of the two edges, see Figure S1.

Figure S8. The plots for A11MoS_2_ for the strains SXYM in (a), SZM in (b), SXYOS in (c), SZOS in (b), SXYIS in (e) and SZIS in (f). Note the significantly large strains at $R=9Å$. This is due to the drastic geometry change of the A11MoS_2_ nanoribbon, especially around the two edges, see Figure S9.


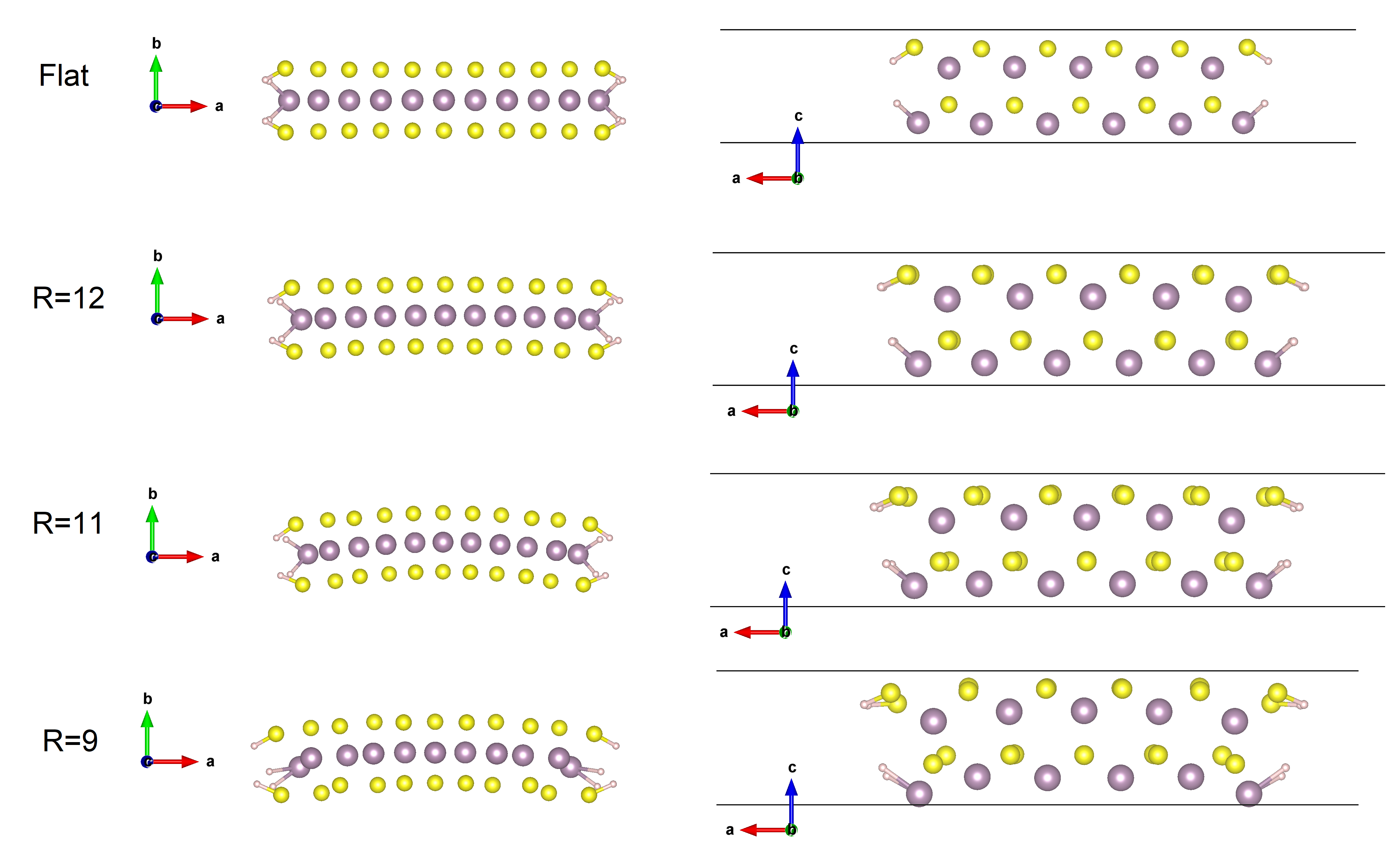


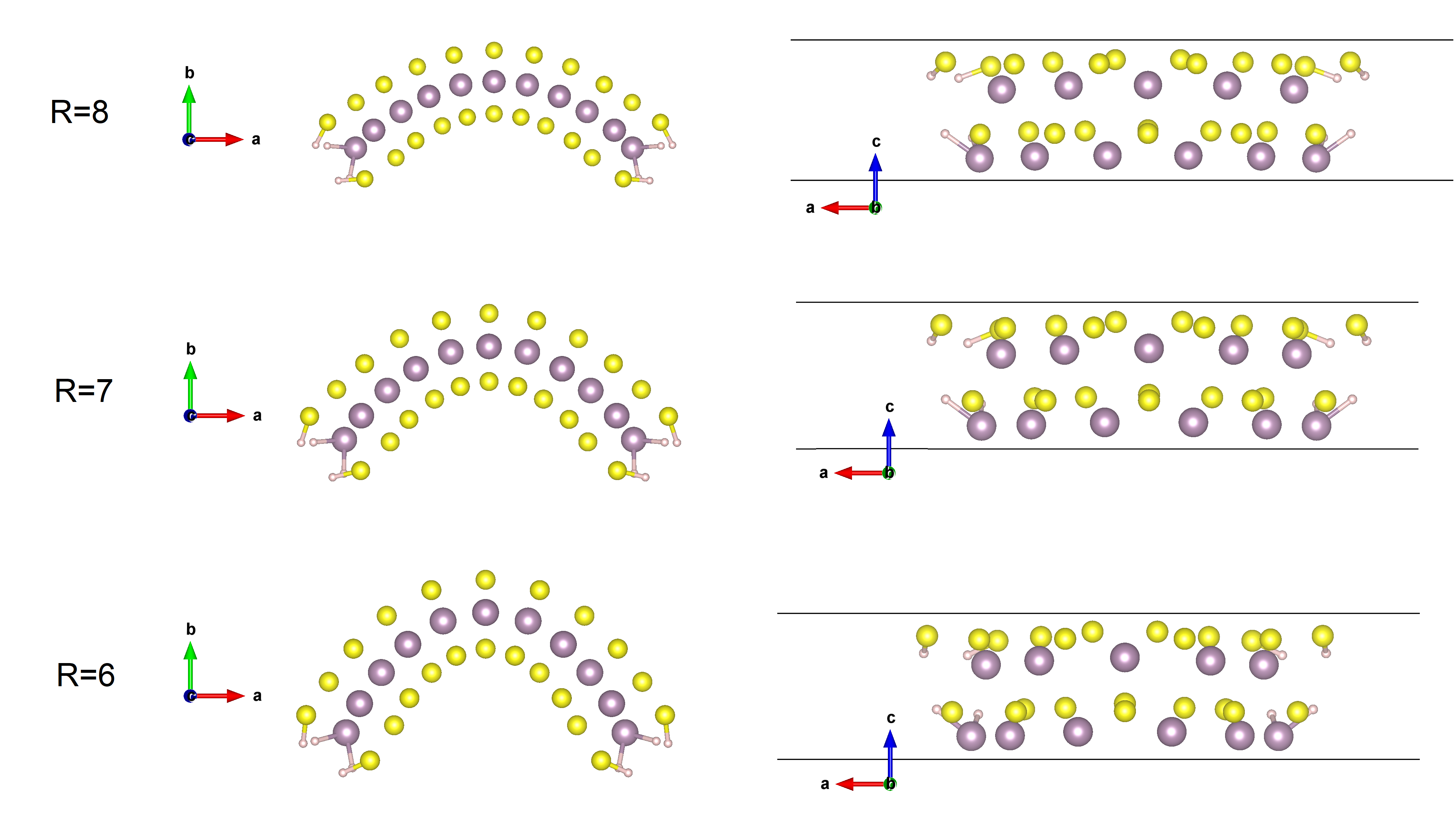


Figure S9. The geometric structures of A11MoS_2_ nanoribbon under different curvature radii. Note the drastic geometry change of the A11MoS_2_ nanoribbon at $R=9Å$, especially around the two edges. This causes the large increase of EG, see Figure S3b, large LC, see Figure S4b, and large strains, see Figure S8.


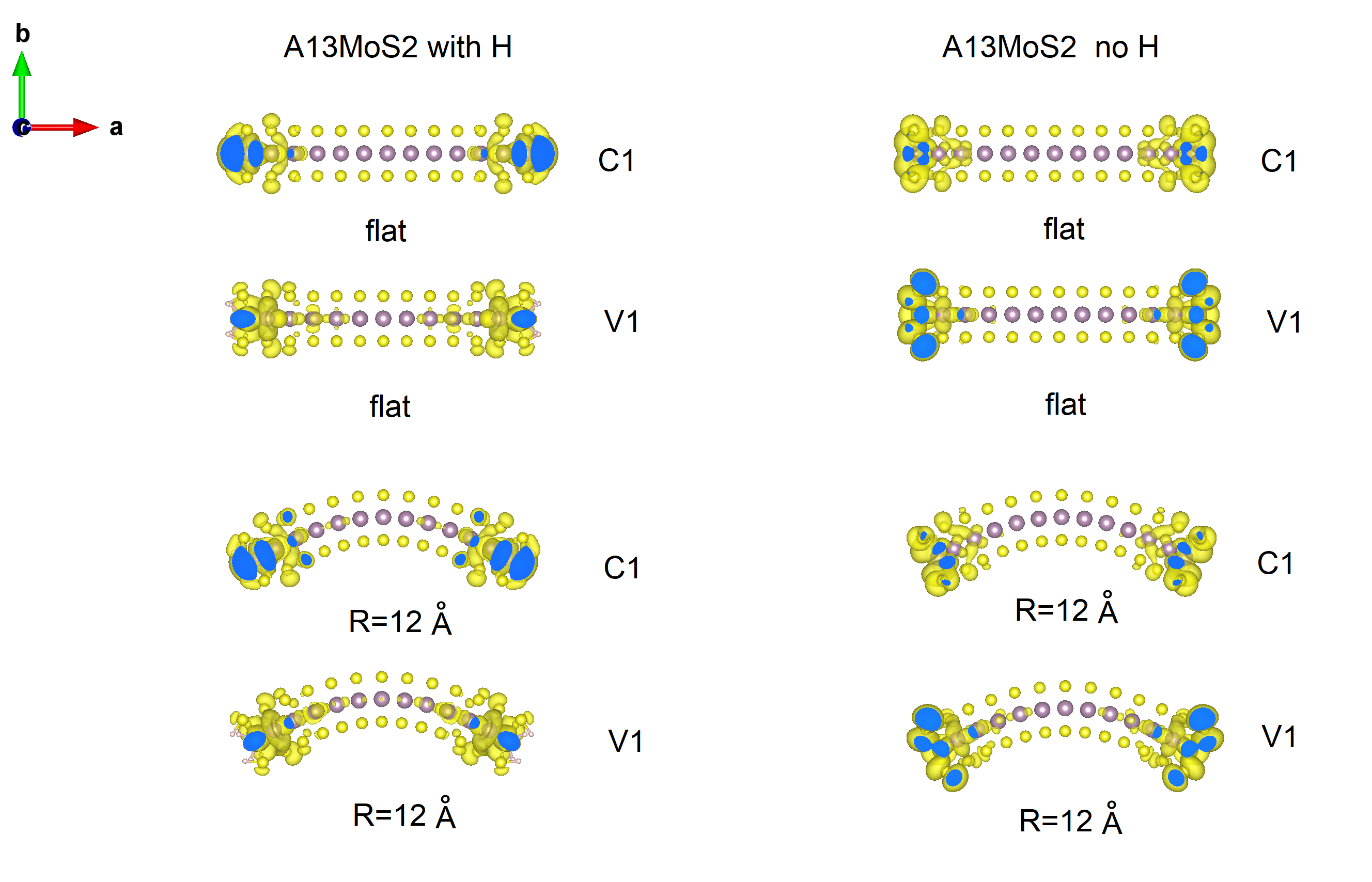


Figure S10. The modulus square of wavefunction of the C1 conduction band and V1 valence band of A13MoS_2_ nanoribbon under bending curvature radius $R=\infty$ (flat) and $R=12Å$ for with hydrogen passivation (the left column) and without hydrogen passivation (the right column). The isosurface value is 0.001 e/Bohr^3^. The hydrogen passivation at the two edges of the nanoribbon increases the extent of the wavefunction of the bands, especially for the conduction band C1, which is important for determining the edge band gap (EG). The yellow color indicates the isosurface, while the blue color indicates the cross section caused by the plane of the supercell.


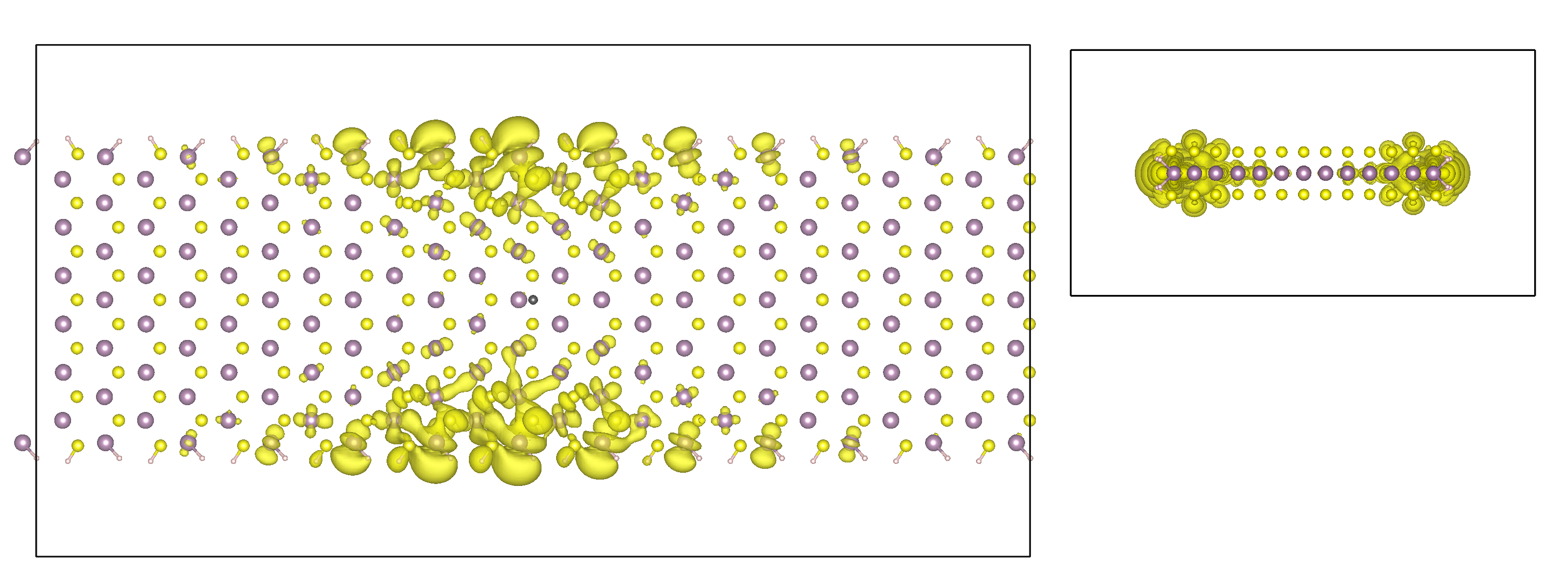


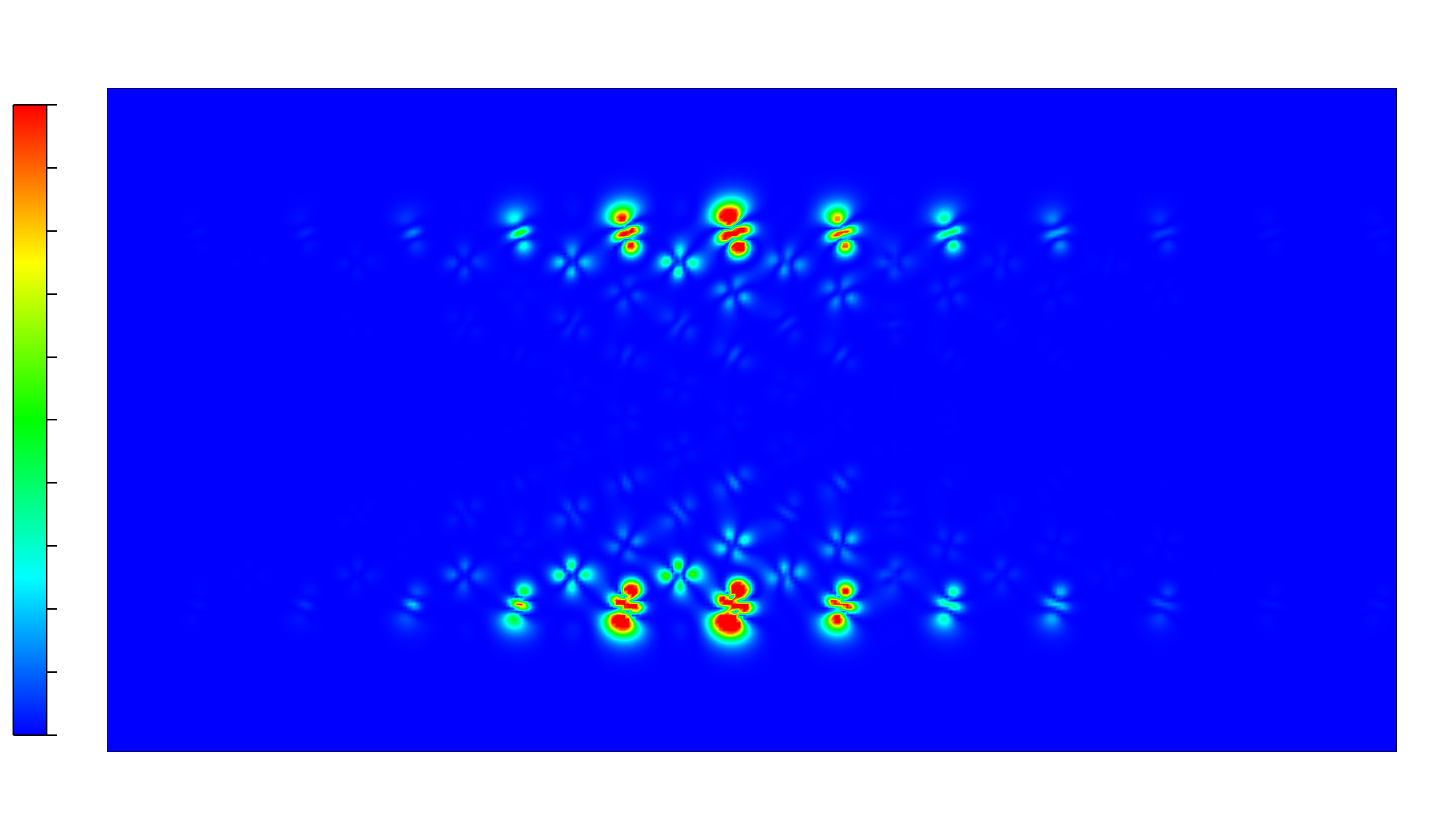

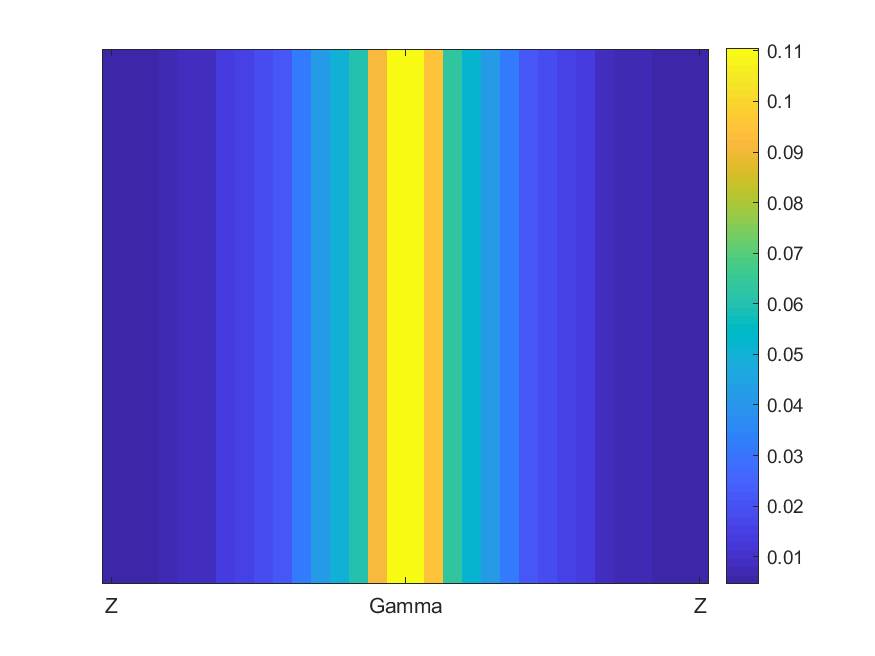


Figure S11. Similar to Figure 6 in the main text, the plots for the exciton forming Peak $B'$ for A13MoS2 at $R=\infty$(flat).


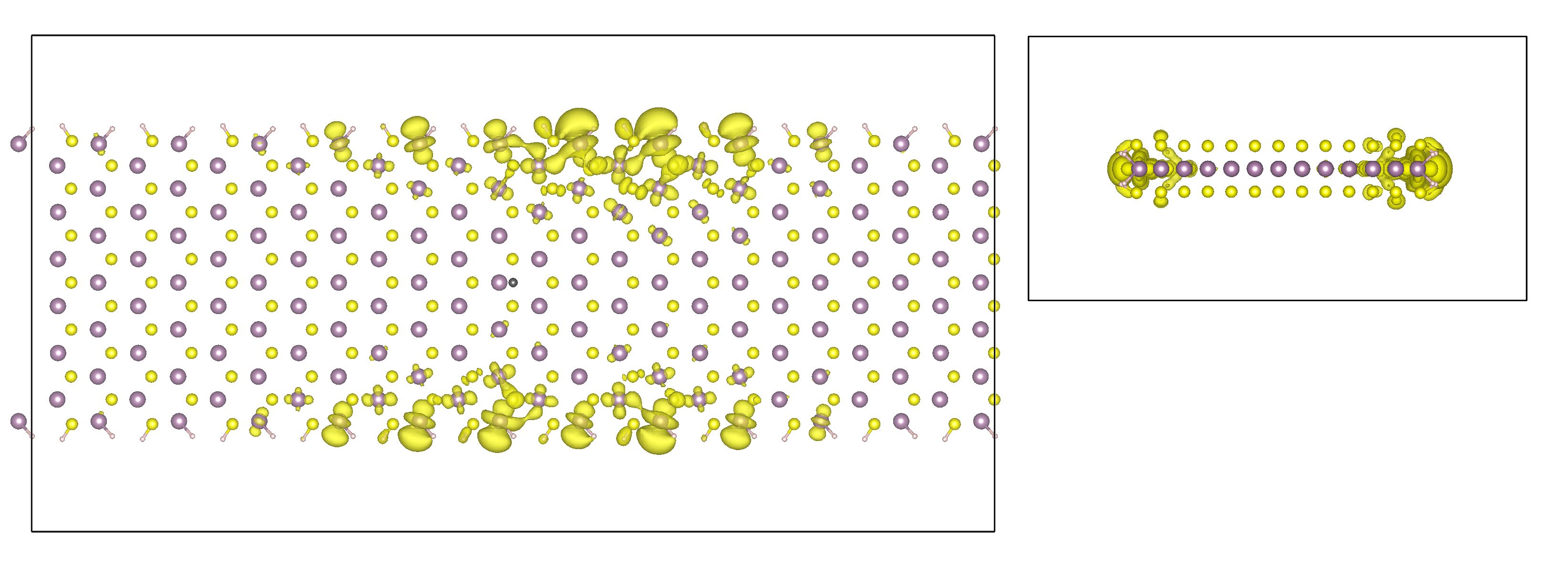


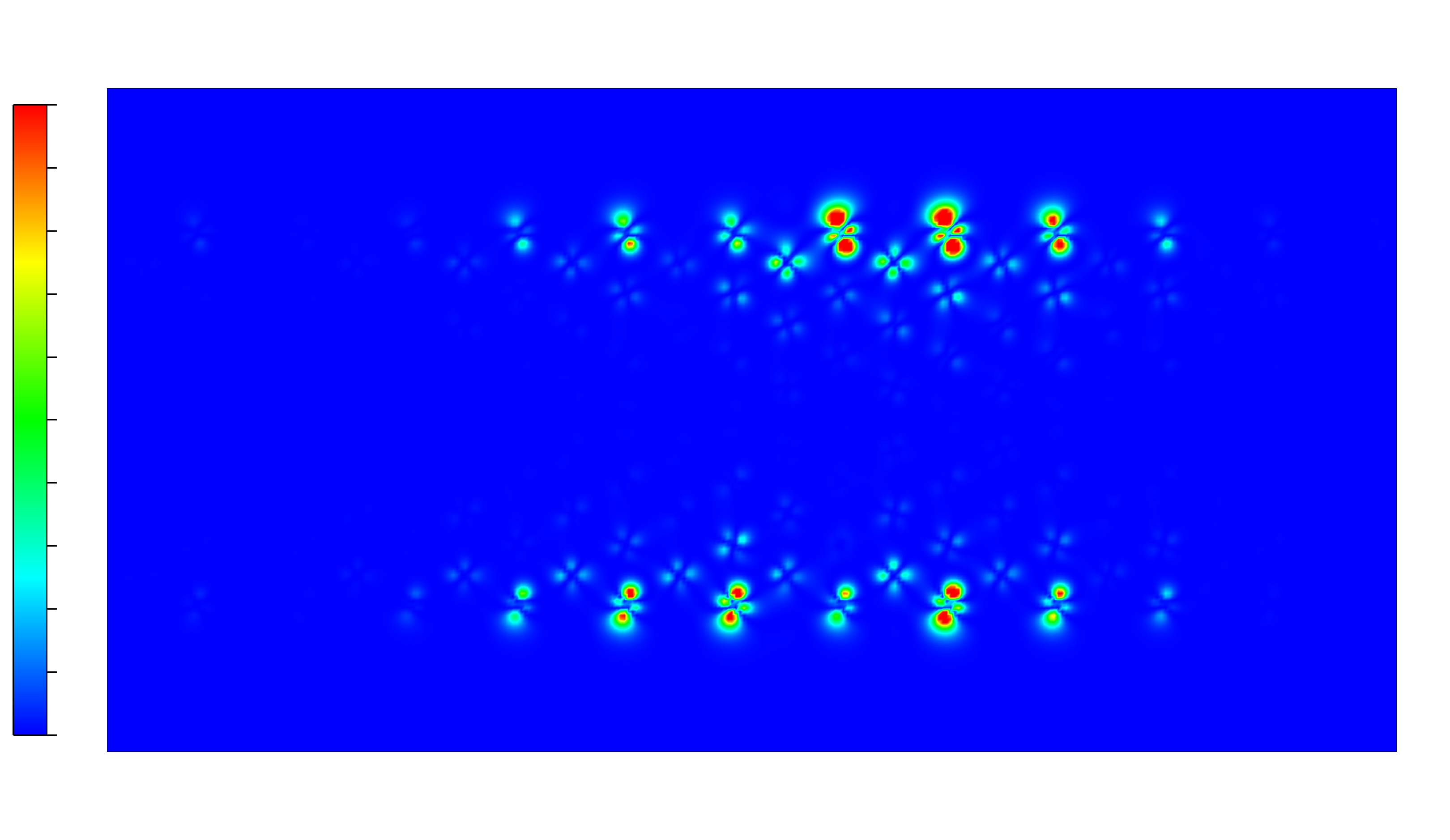

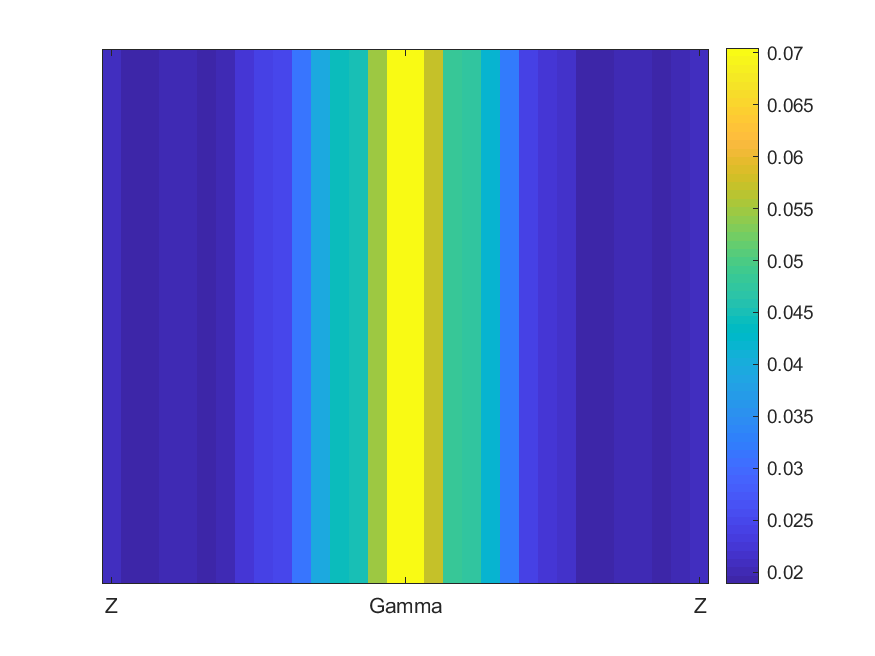


Figure S12. Similarly plotted with Figure 6 in the main text, the plots for the exciton at 1.01 eV for A13MoS2 at $R=\infty$(flat), which is merged in the right bottom of peak $B'$. Note the nodal feature of the wave function. It is the excited state of the exciton forming peak $A'$.


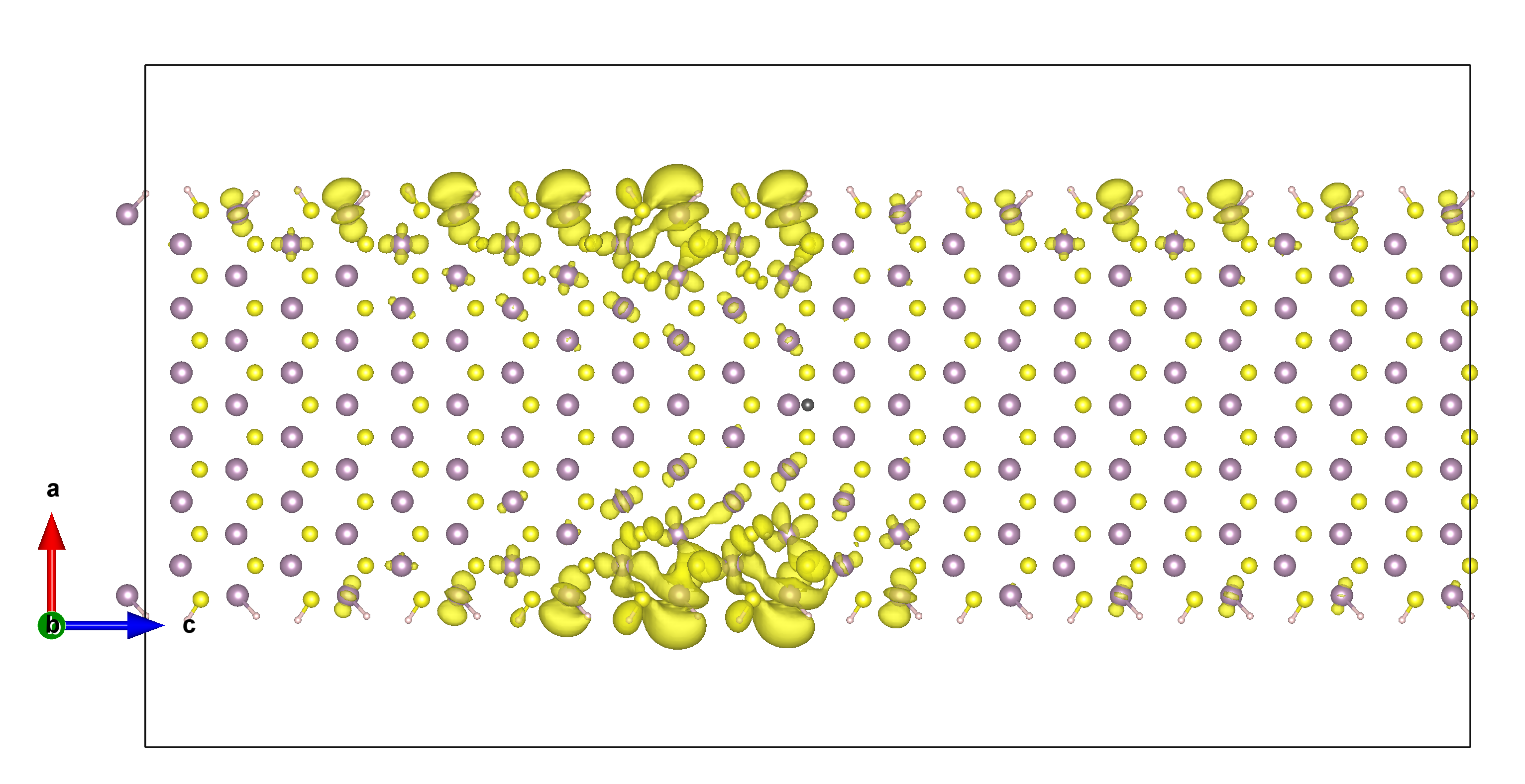

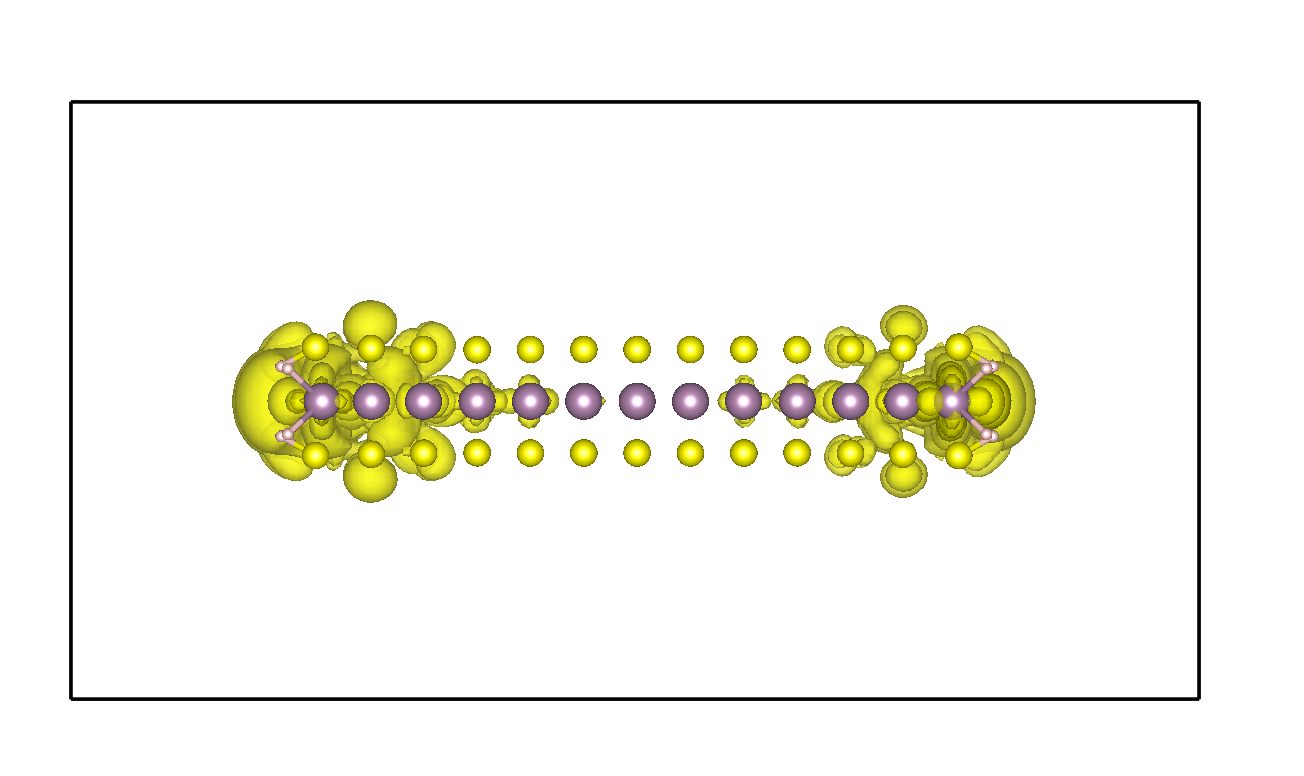


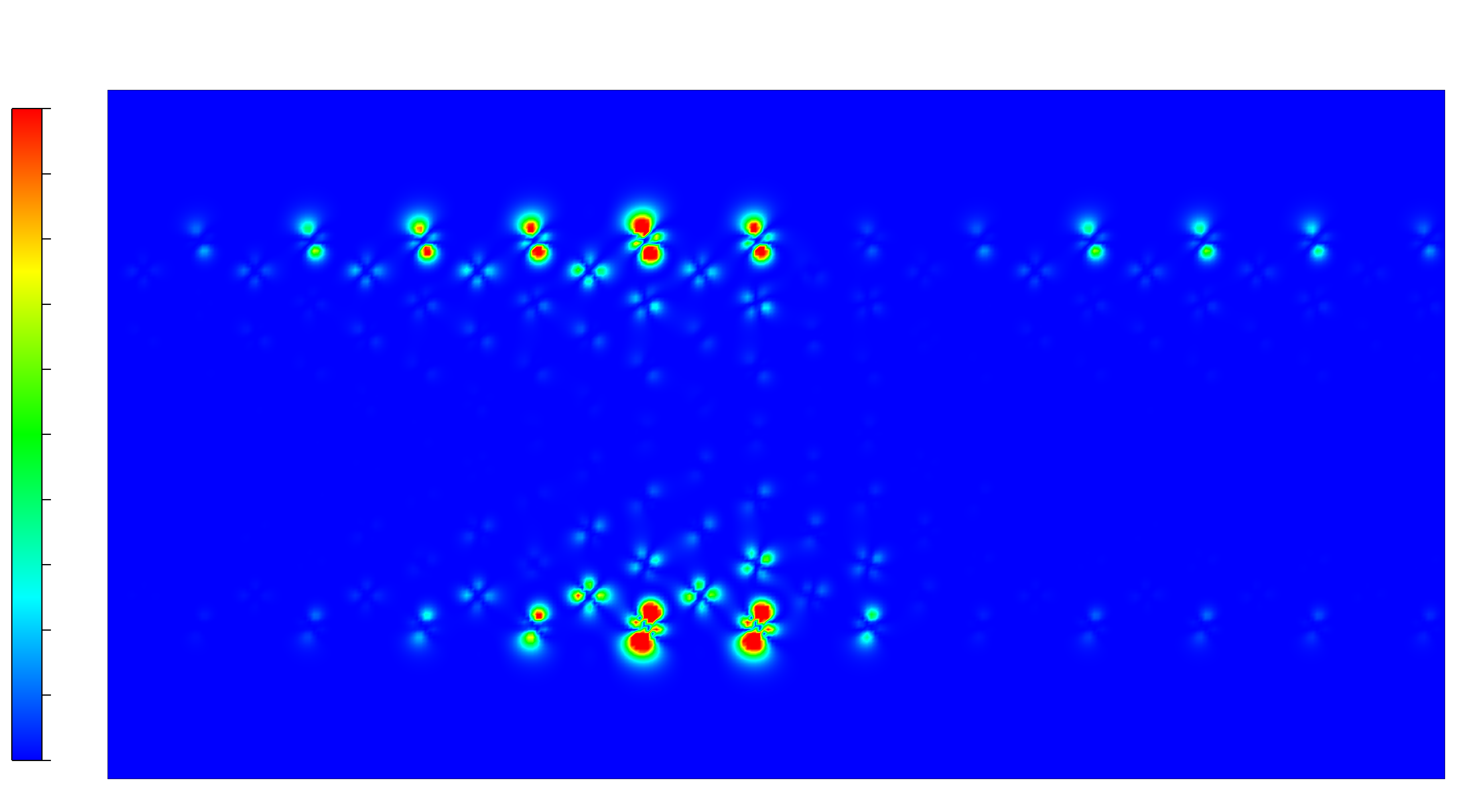

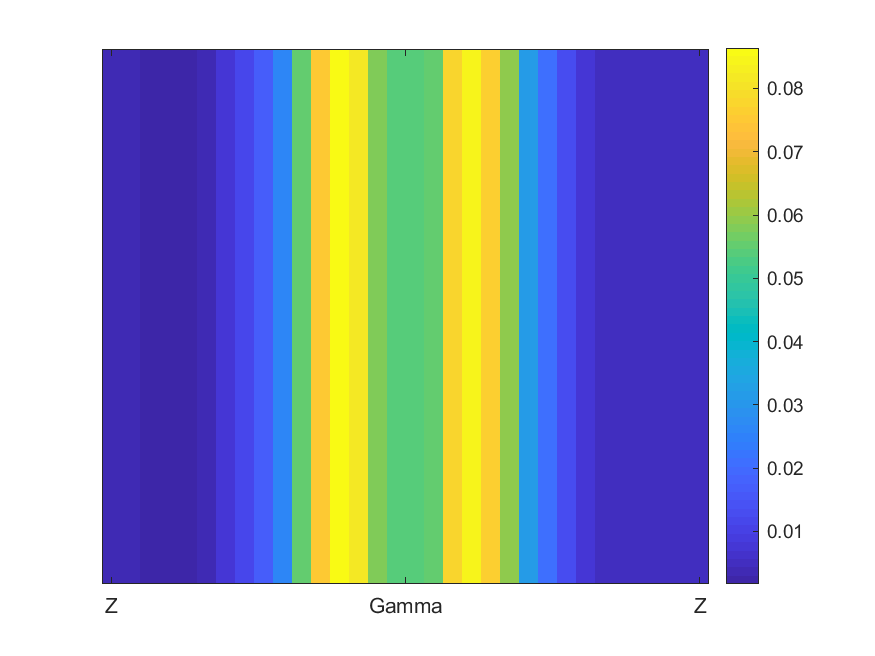


Figure S13. The plots for the exciton at 1.17 eV for A13MoS2 at $R=\infty$(flat), Note the nodal feature of the wave function, indicating that it is the excited state of the exciton forming peak $B'$.


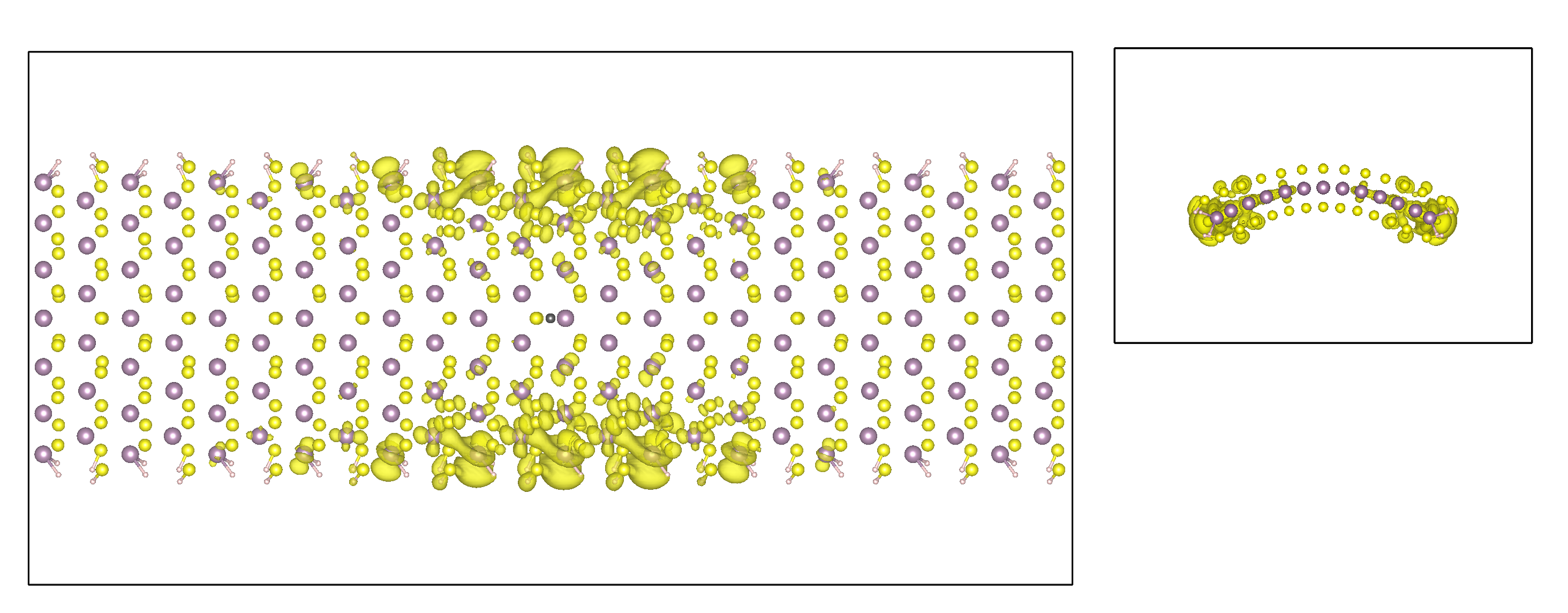


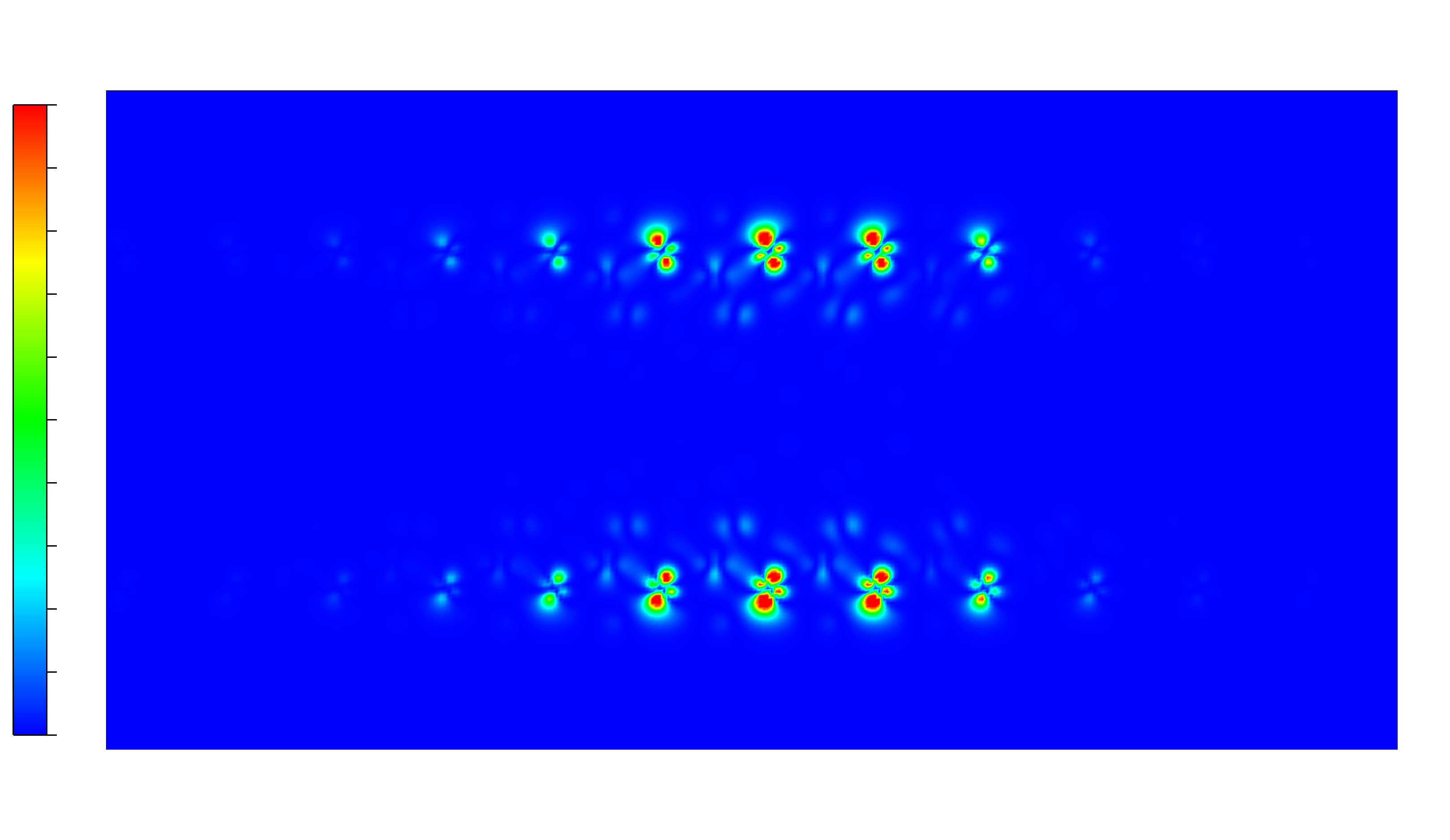

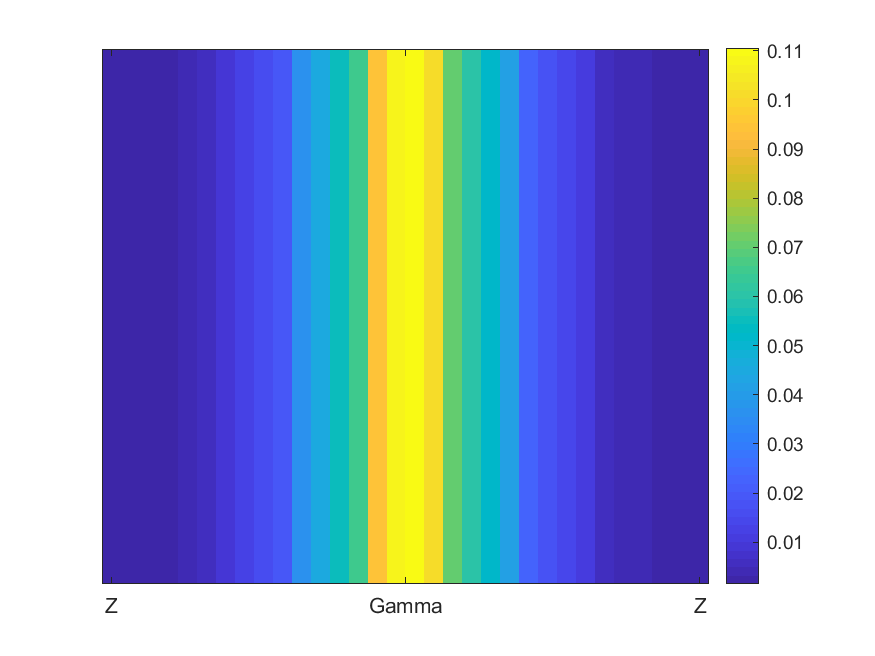


Figure S14. The plots for the exciton forming Peak $A''$ for A13MoS2 at $R=13Å$.


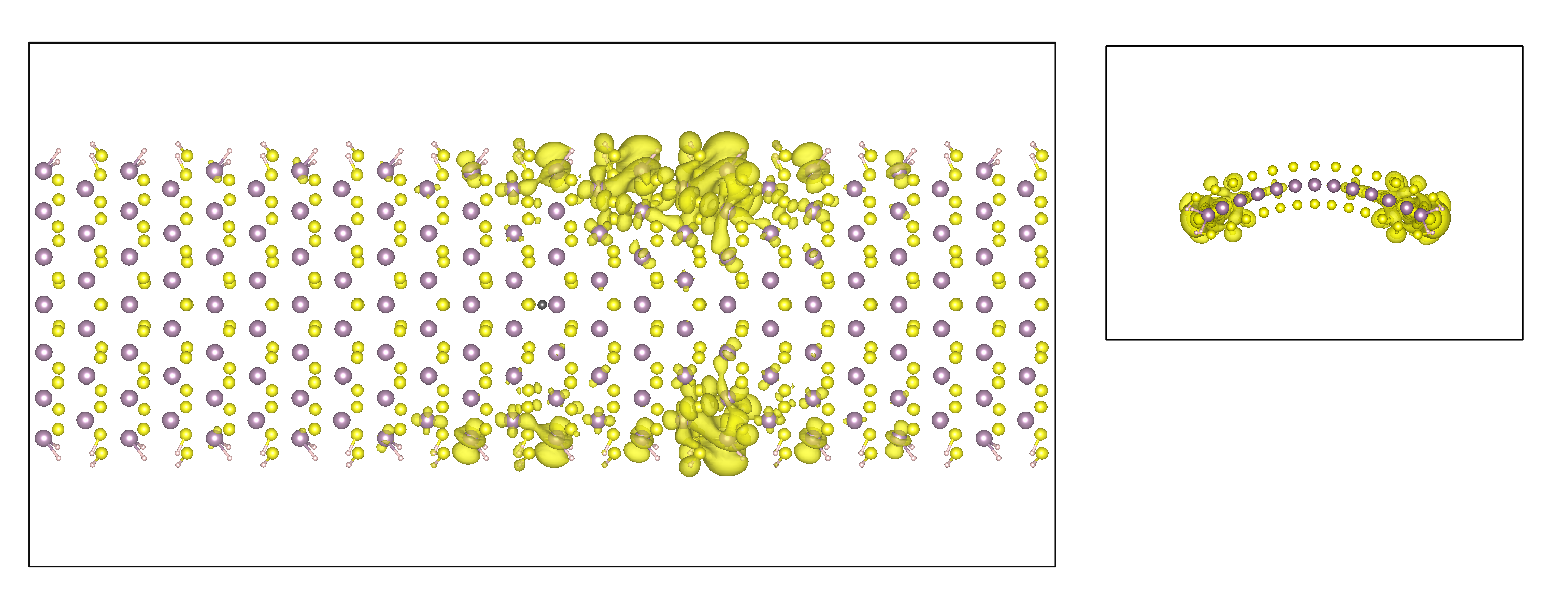


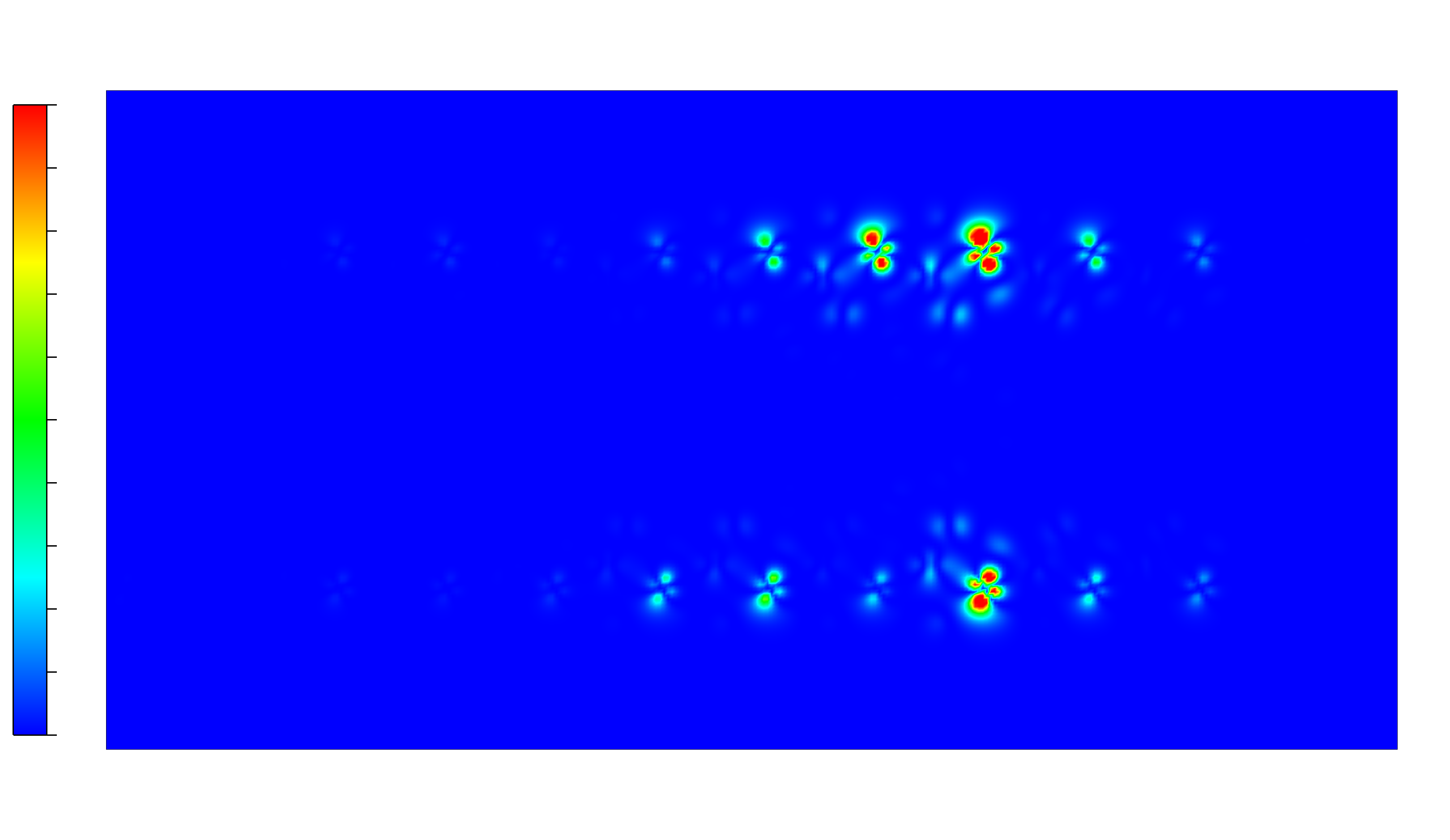

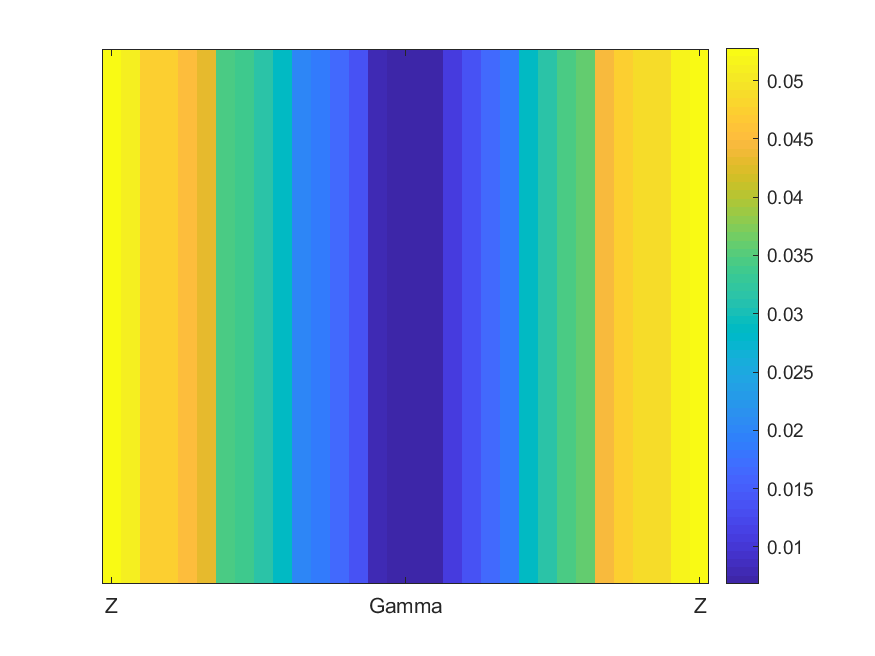


Figure S15. The plots for the exciton at 0.75 eV for A13MoS2 left$R=13Å$, it shows nodal features. It relates to the excited state of exciton forming peak $A''$.


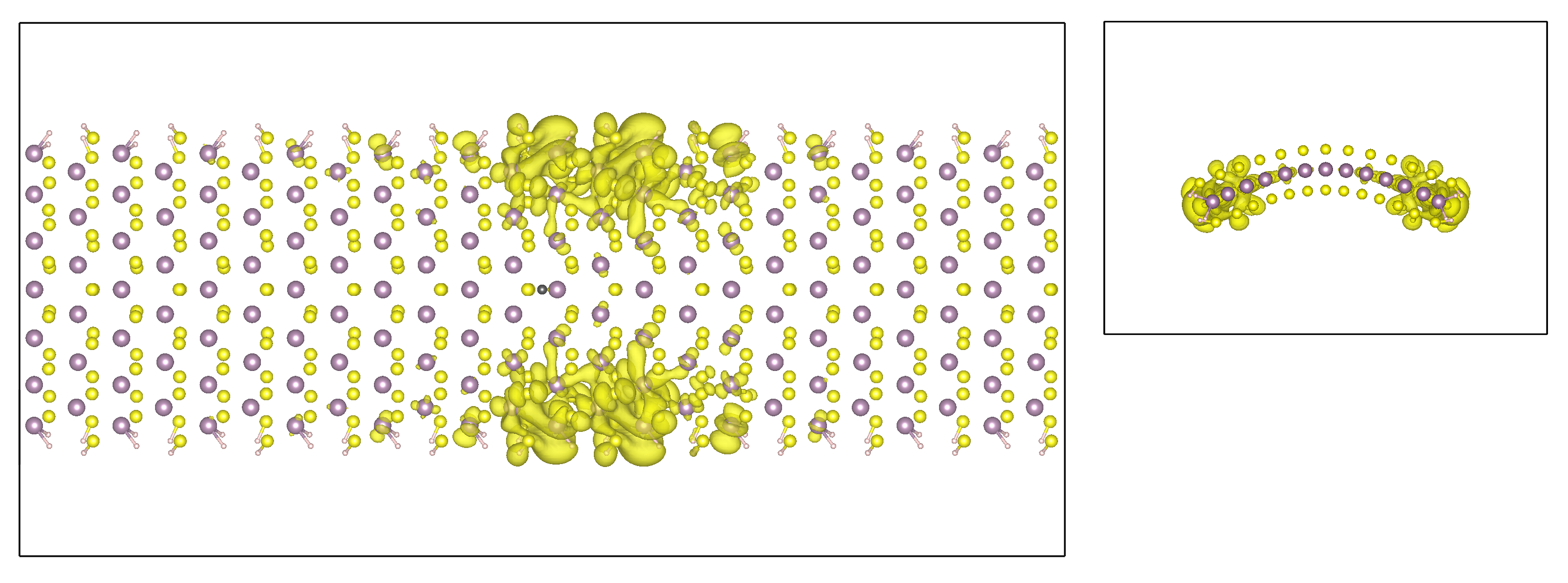


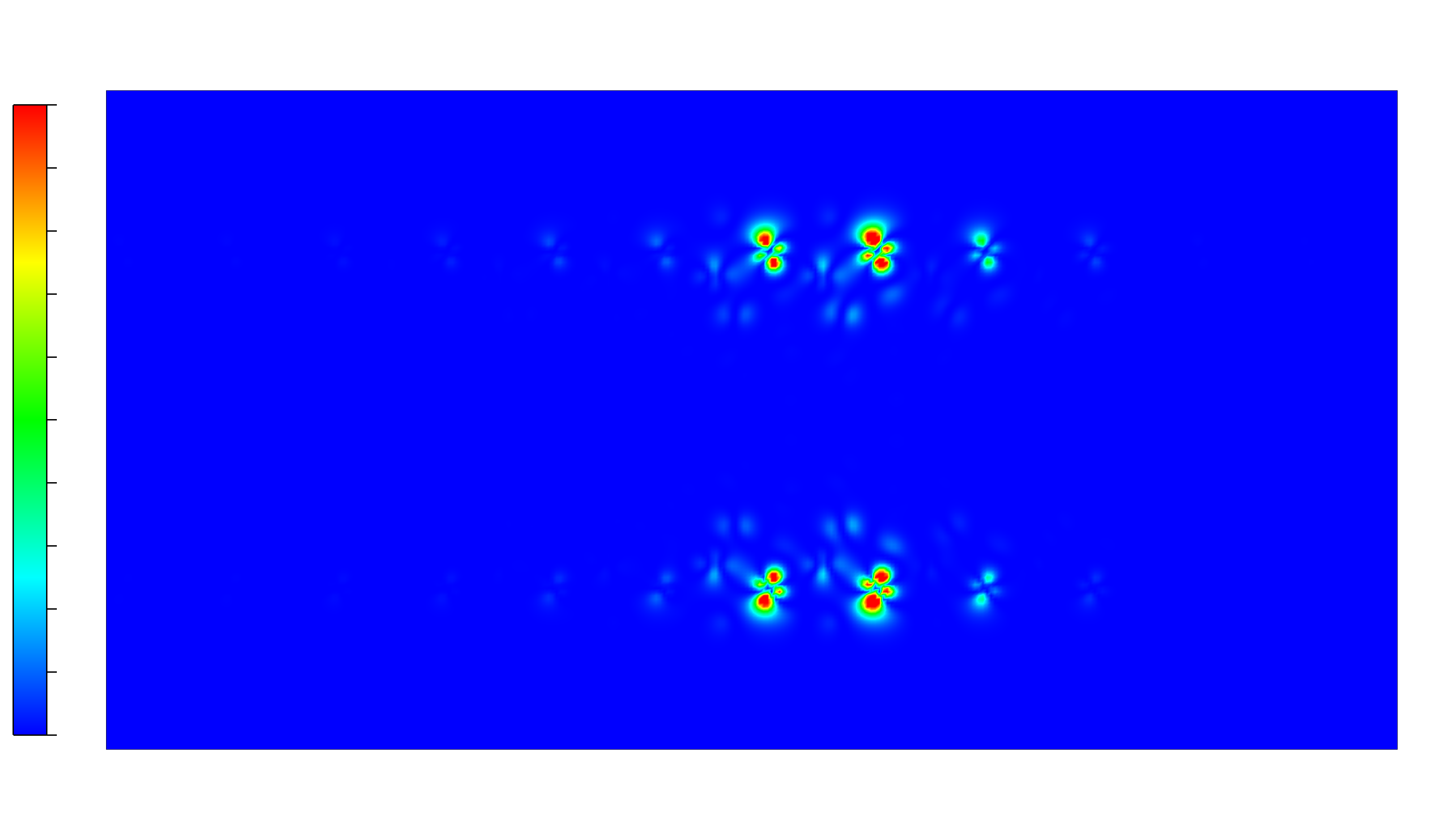

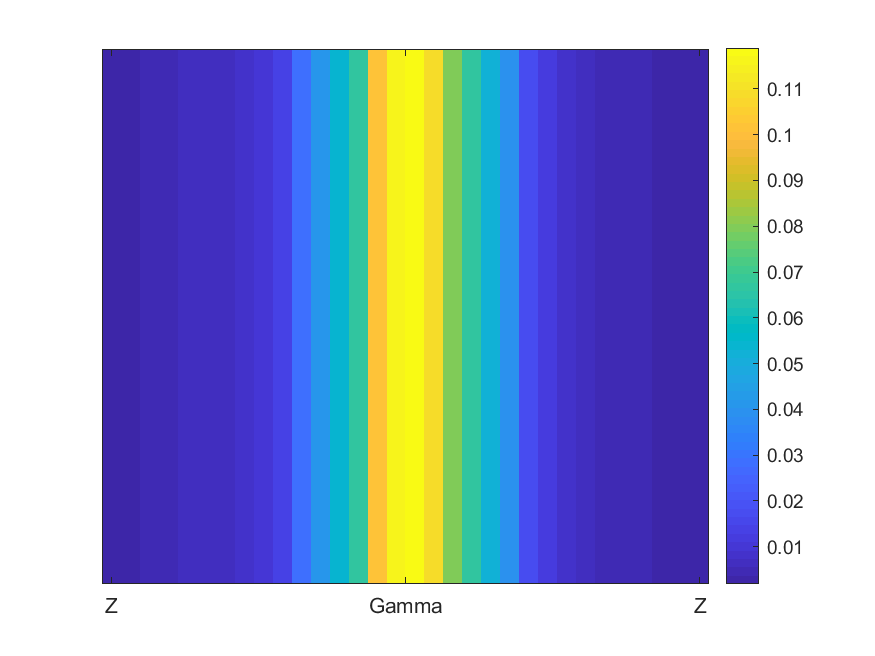


Figure S16. The plots for the exciton forming Peak $B''$ for A13MoS2 at $R=13Å$. It relates to transitions around $\Gamma$ from V3 to C1 (or V3 to C2).


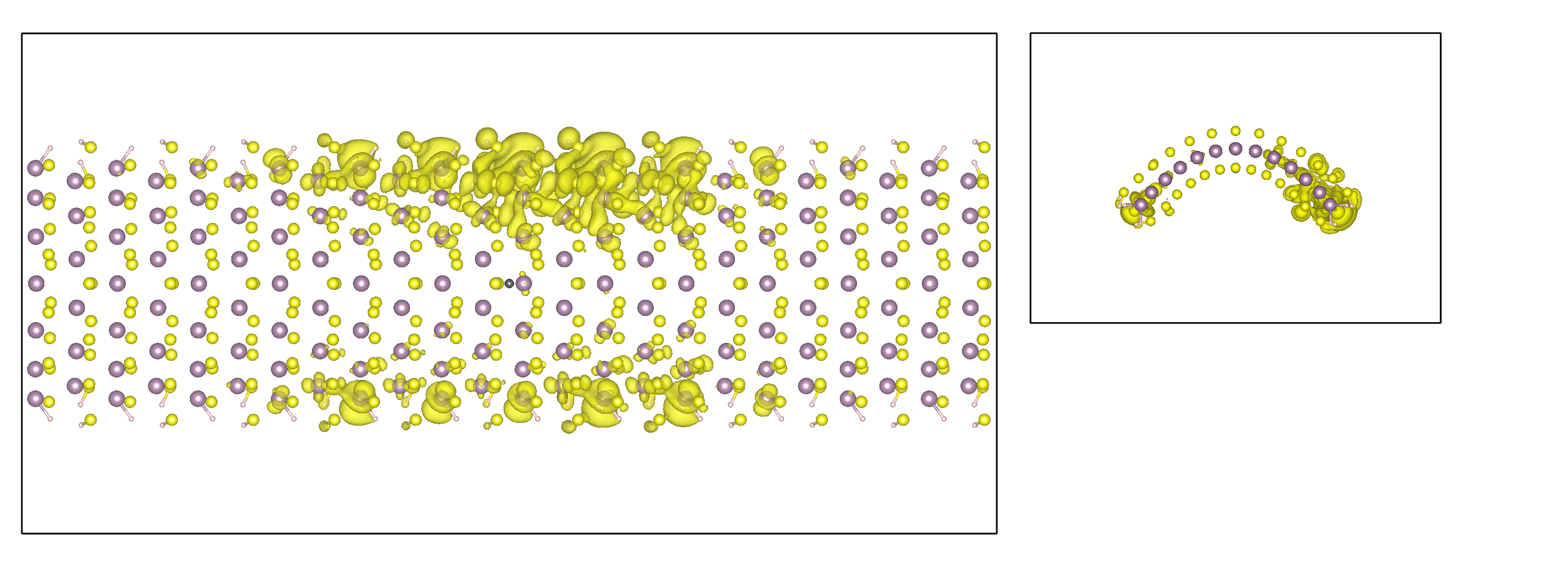


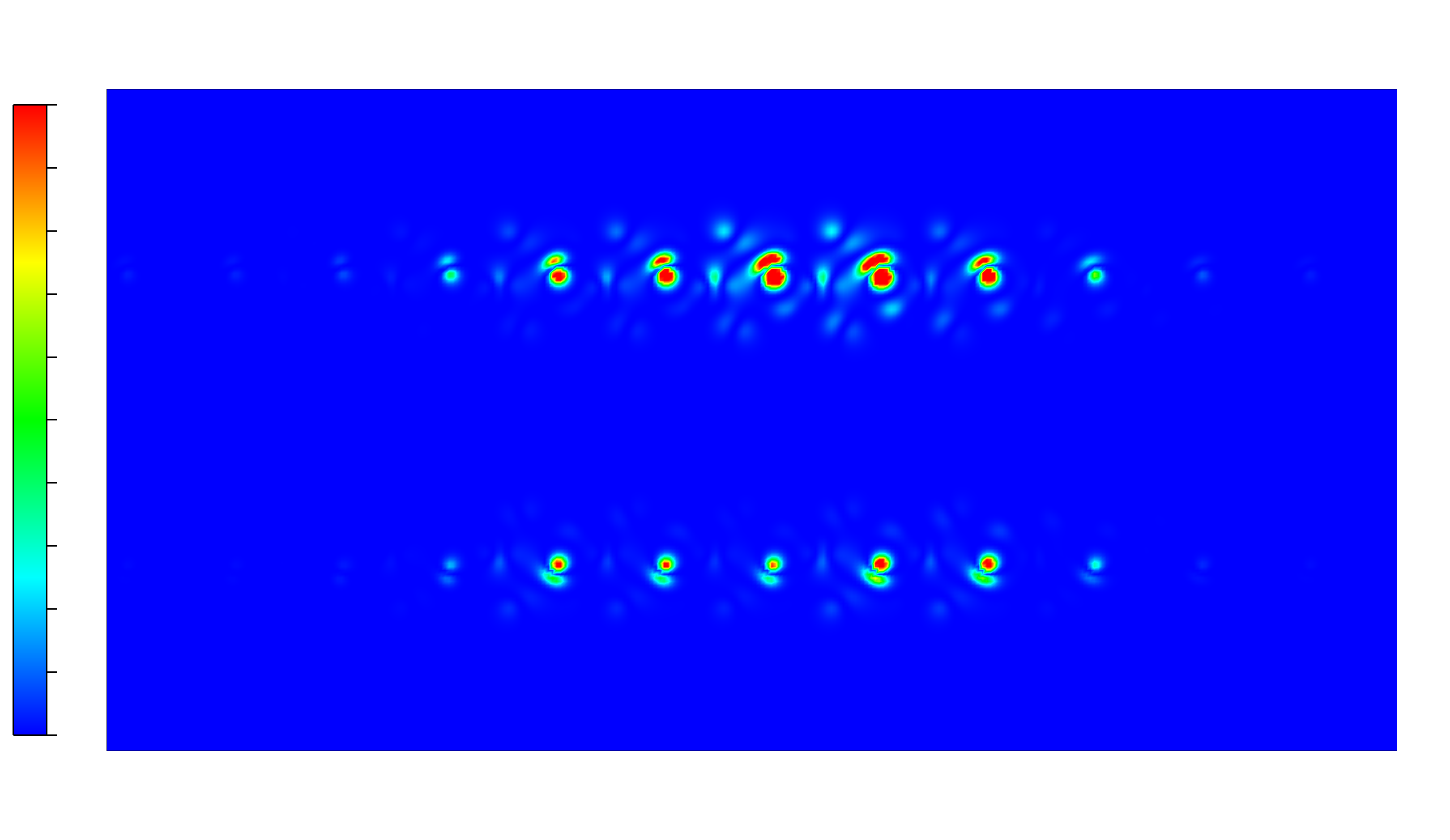

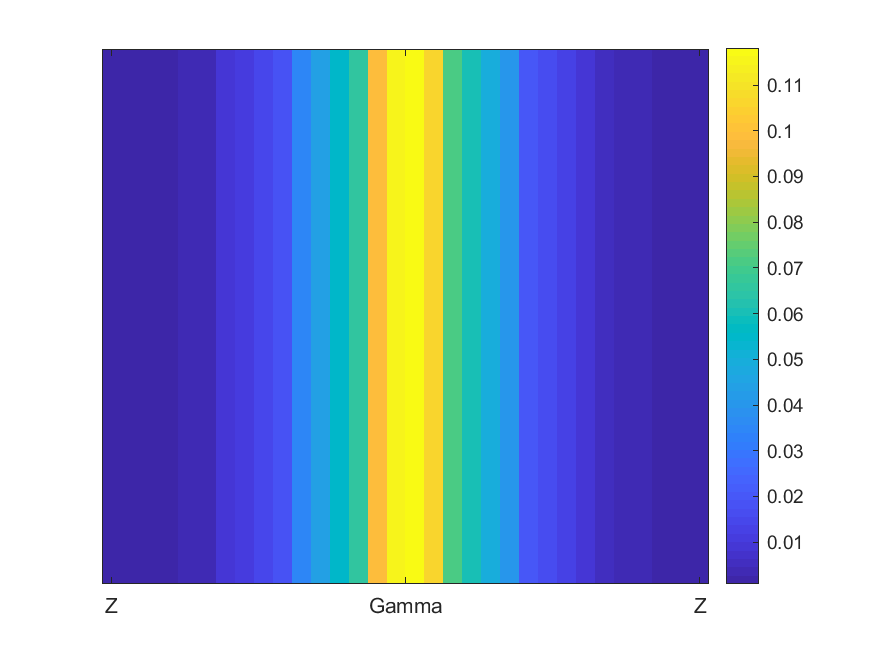


Figure S17. The plots for the exciton forming Peak $A'''$ for A13MoS2 at $R=9Å$.


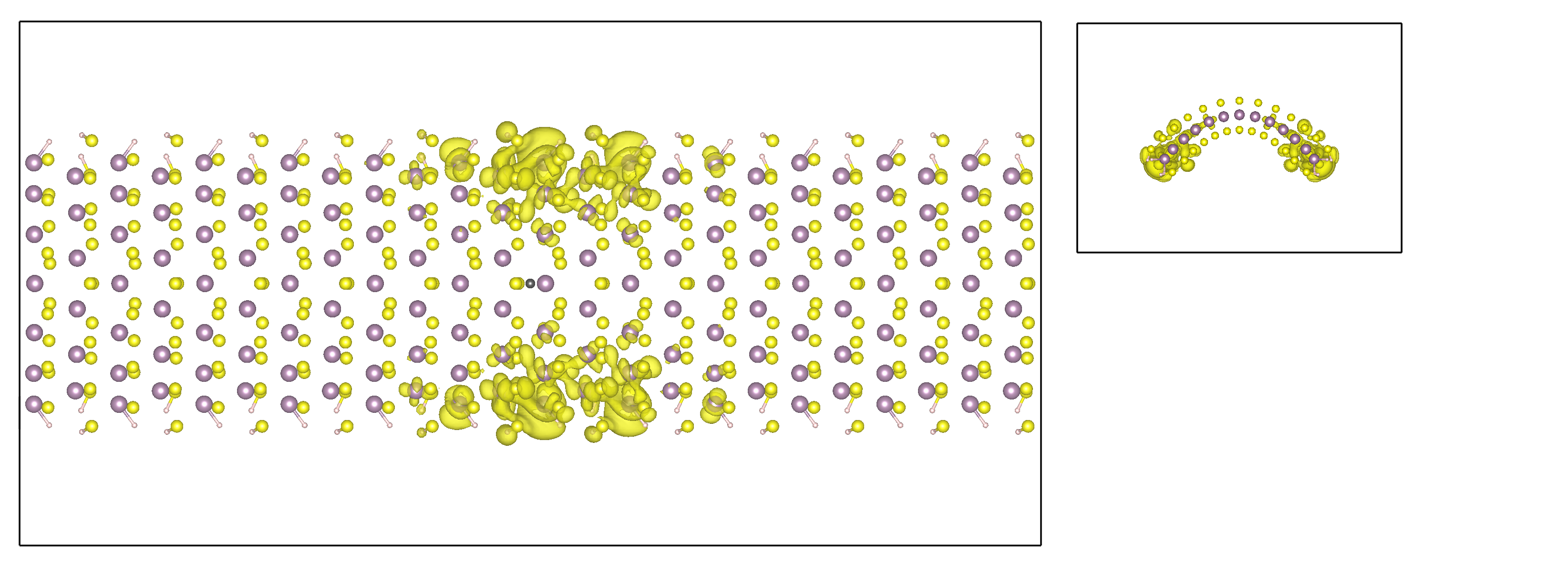


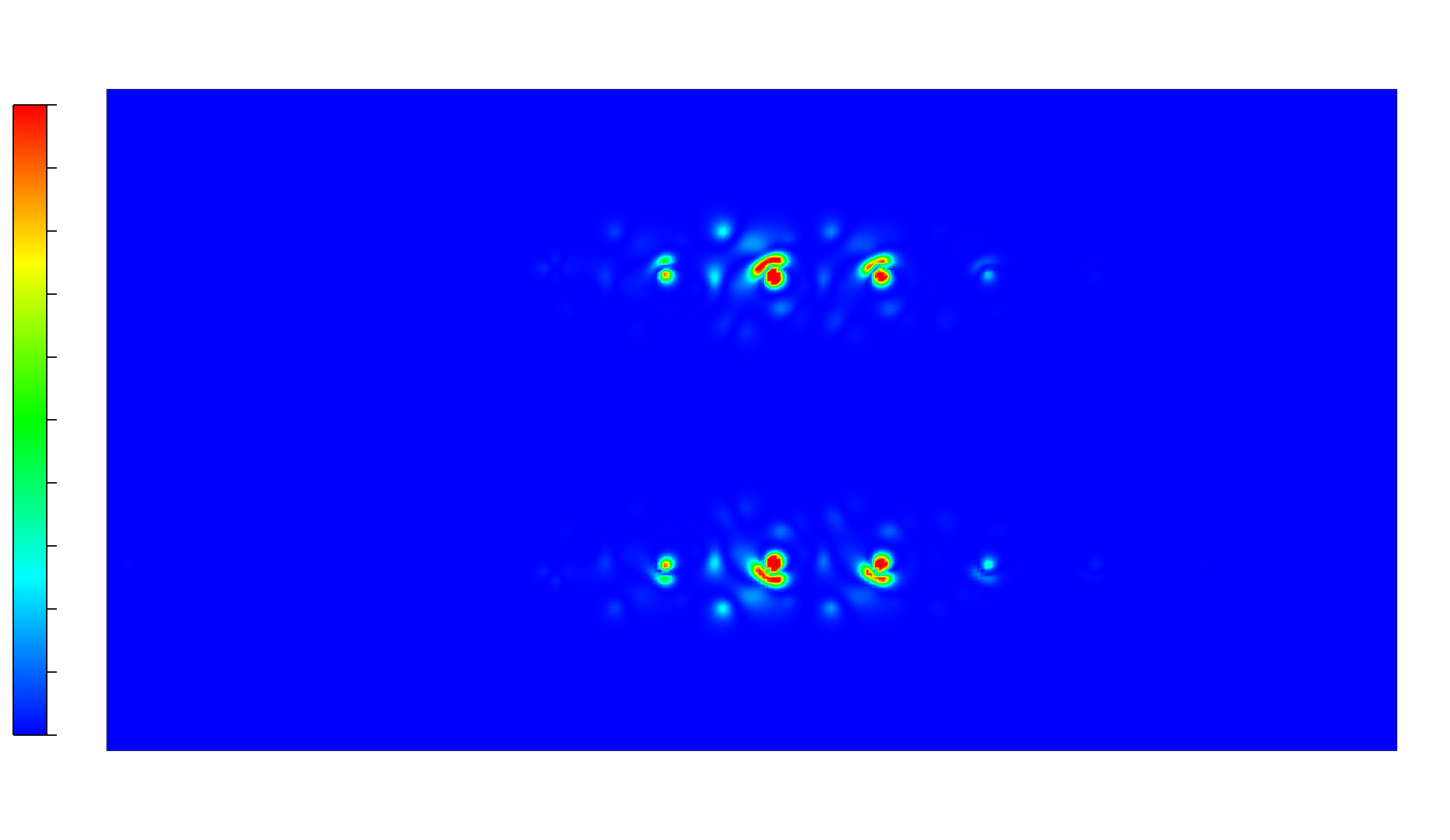

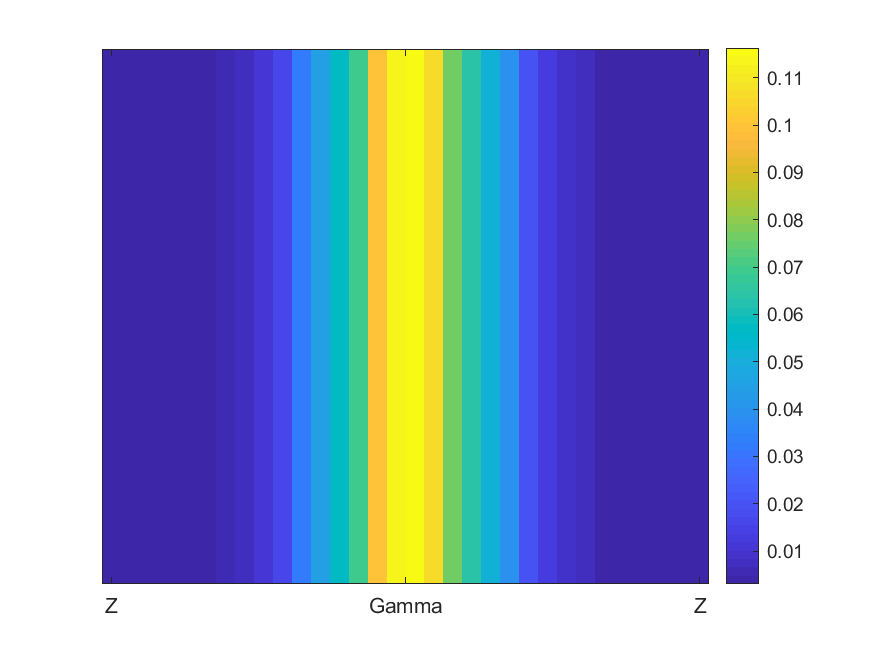


Figure S18. The plots for the exciton at 0.65 eV for A13MoS2 at $R=9Å$. It relates to transitions V1$\to$C1/C2.


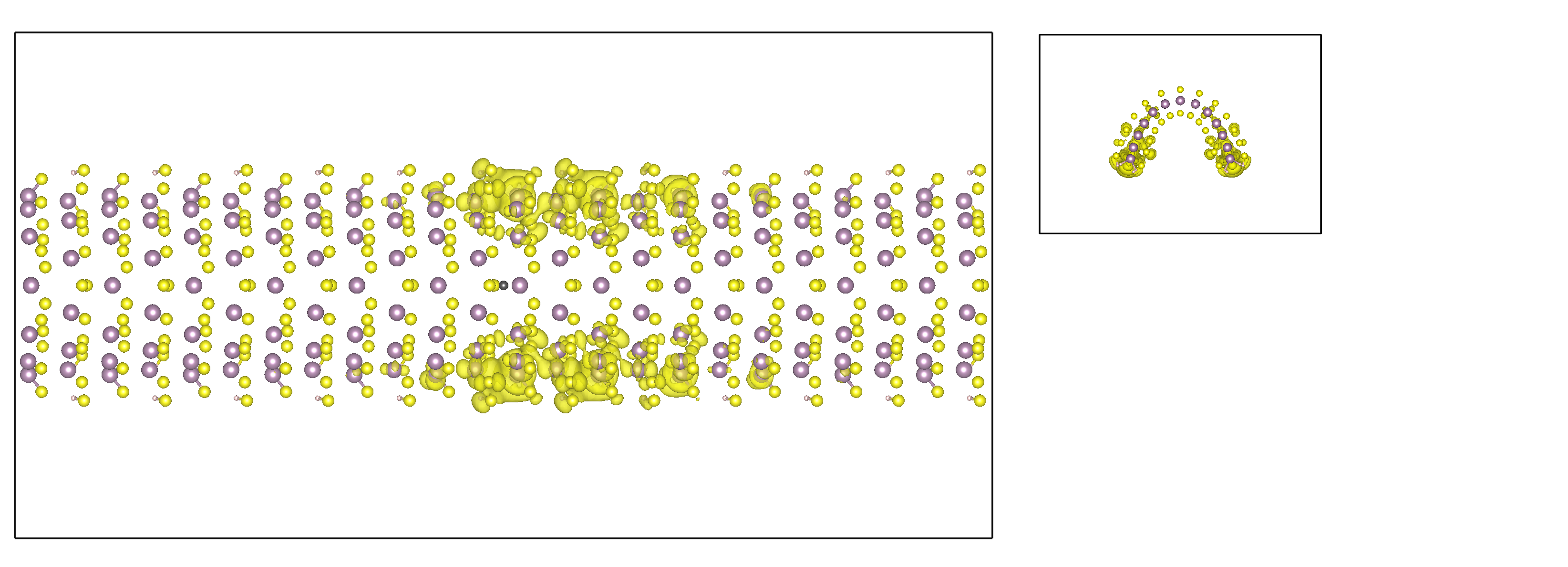

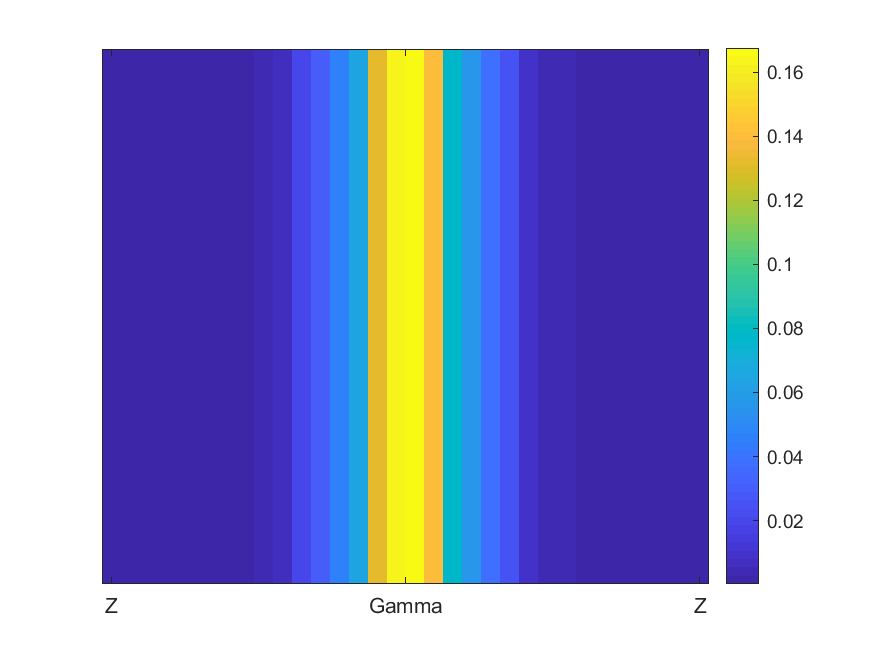


Figure S19. The plots for the exciton forming peak $A''''$ for A13MoS2 at $R=6Å$.


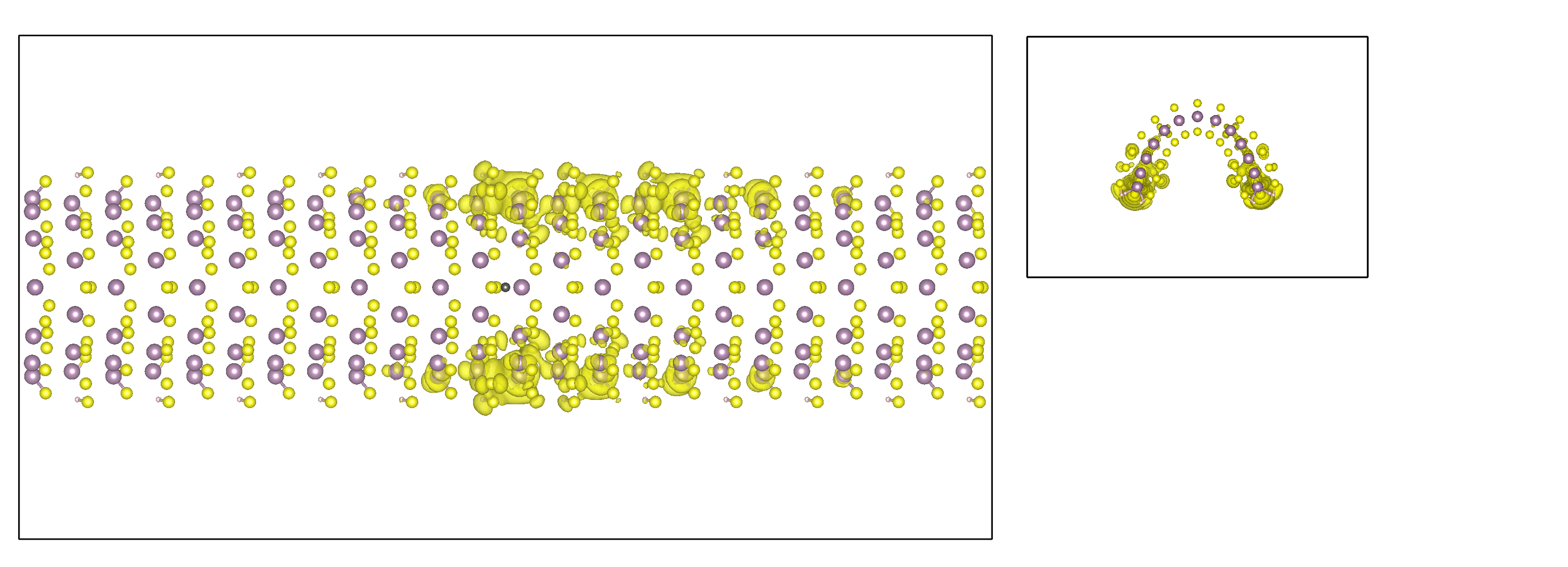

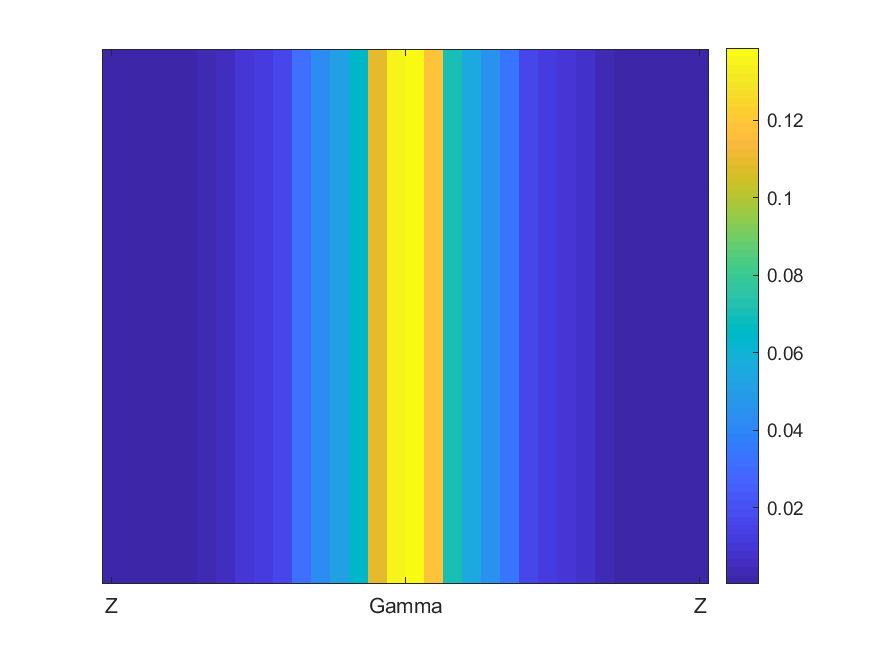


Figure S20. The plots for the exciton forming peak $B''''$ for A13MoS2 at $R=6Å$.


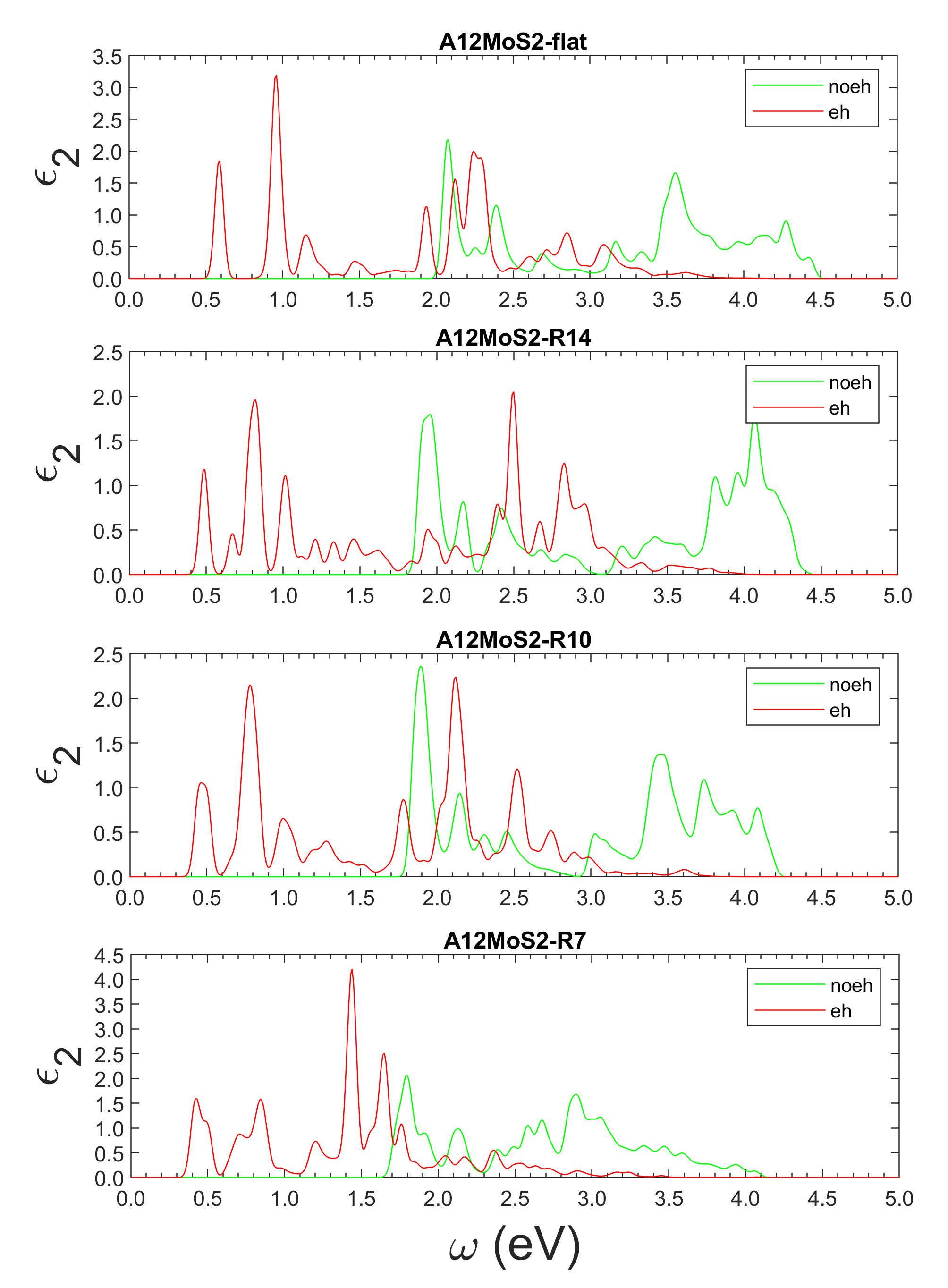


Figure S21. The optical absorption spectra of A12MoS_2_ nanoribbon under different curvature radii from GW+BSE calculations.


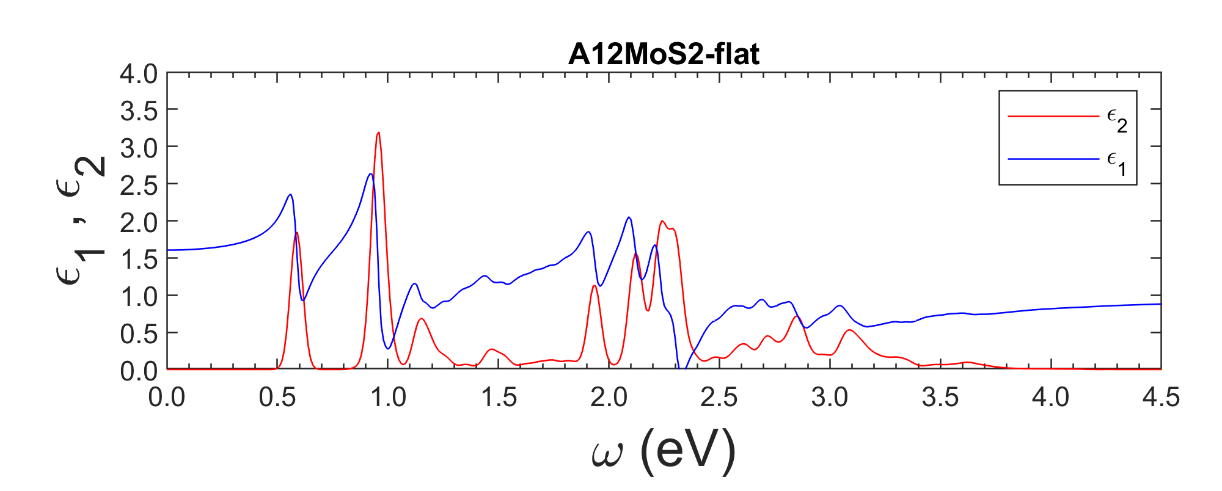


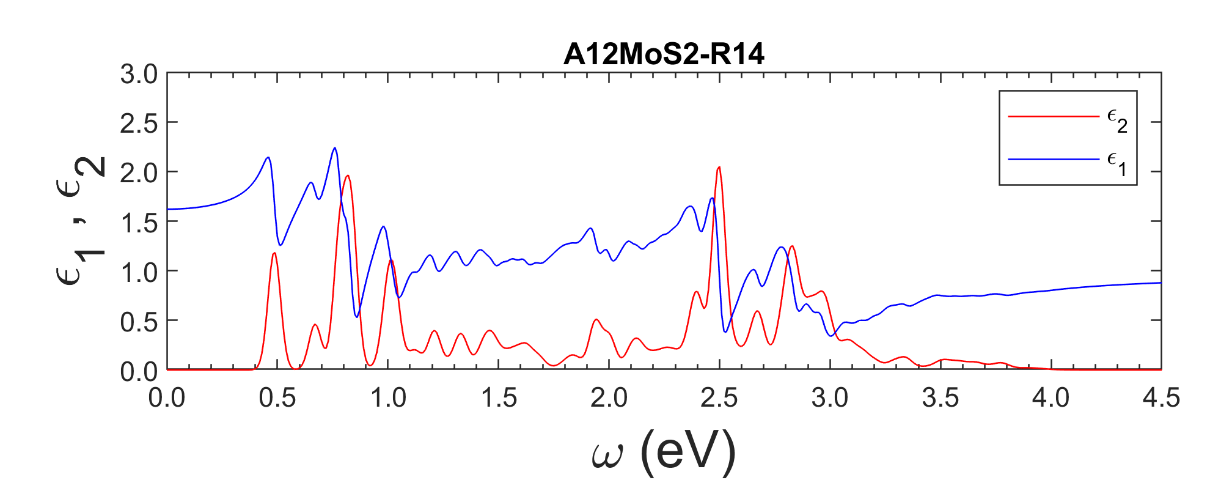


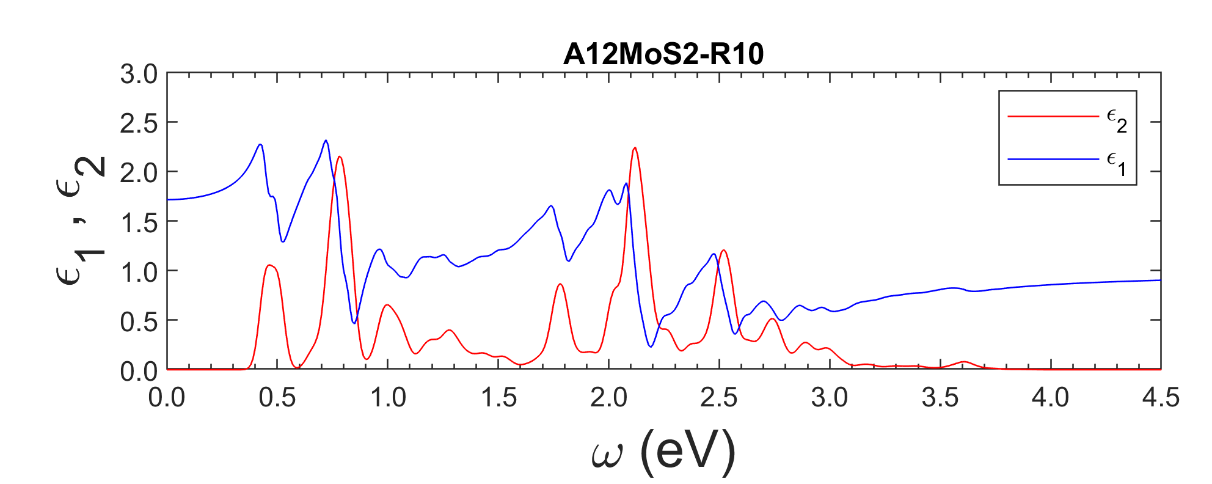


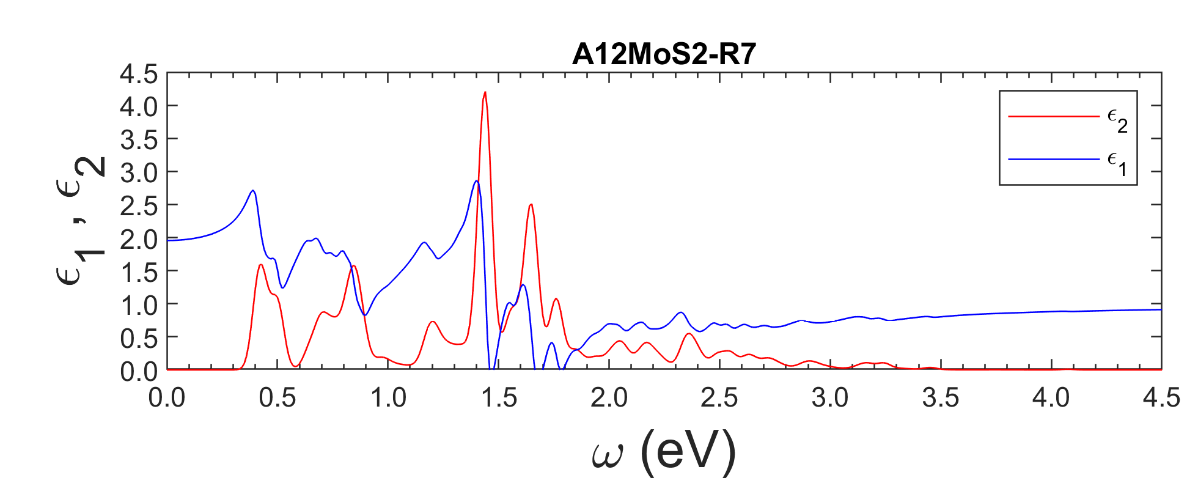


Figure S22. The real and imaginary parts of the dielectric function vs. frequency under different bending curvatures for A12MoS_2_ nanoribbon.


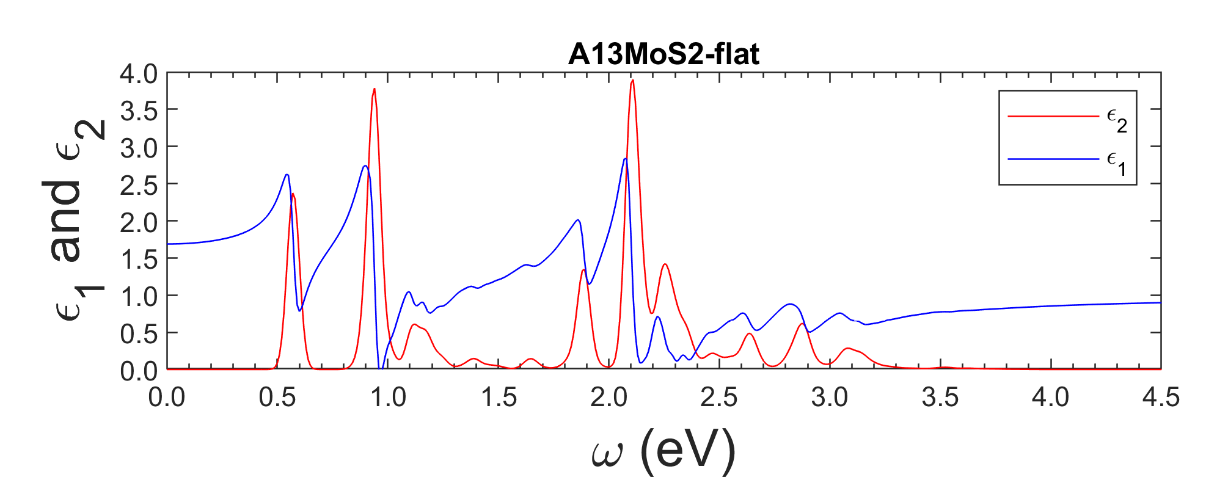


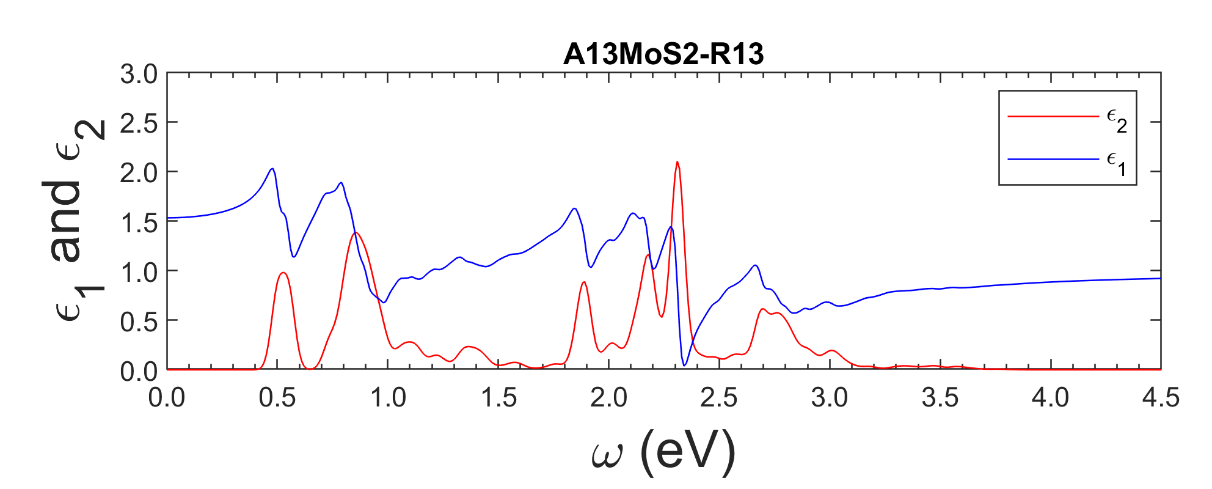


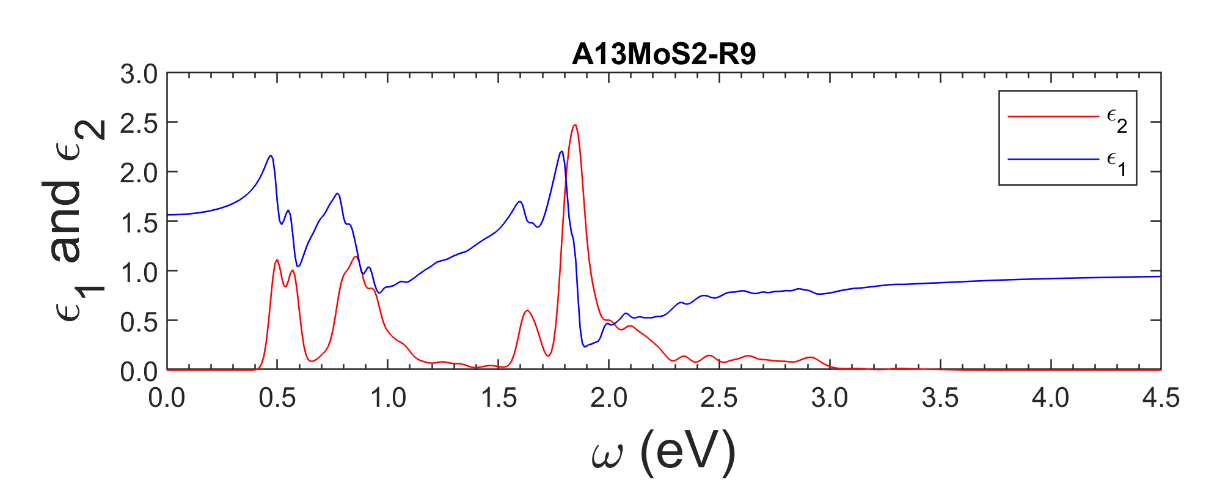


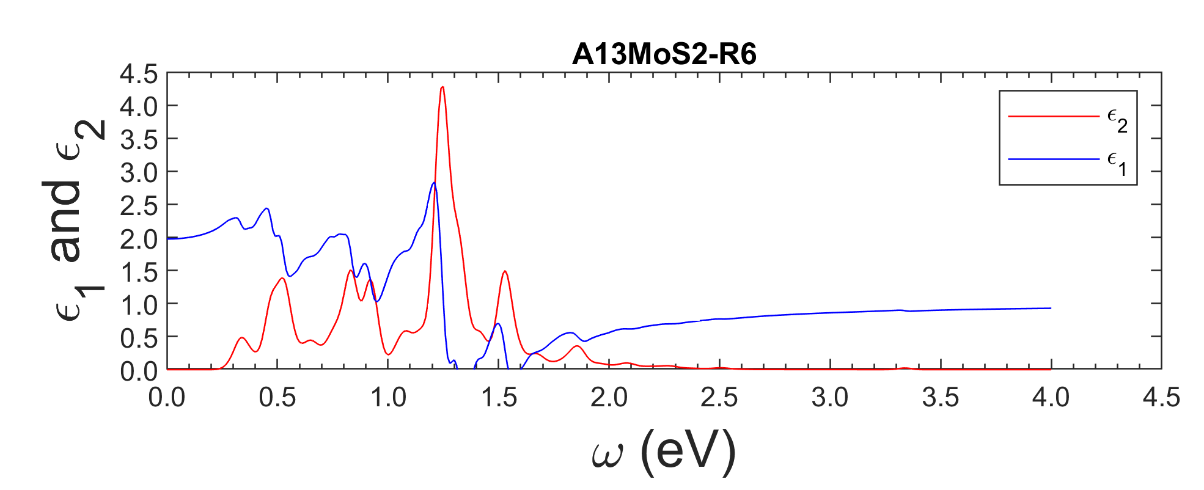


Figure S23. The real and imaginary parts of the dielectric function vs. frequency under different bending curvatures for A13MoS_2_ nanoribbon.


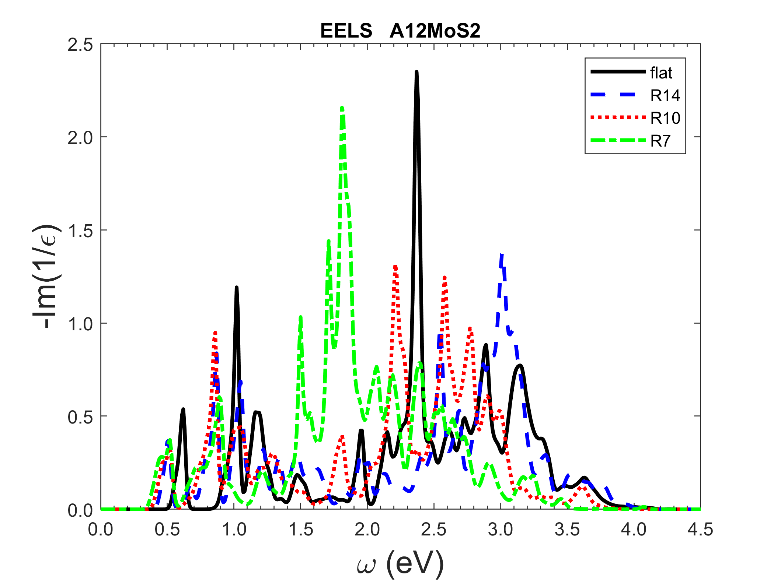

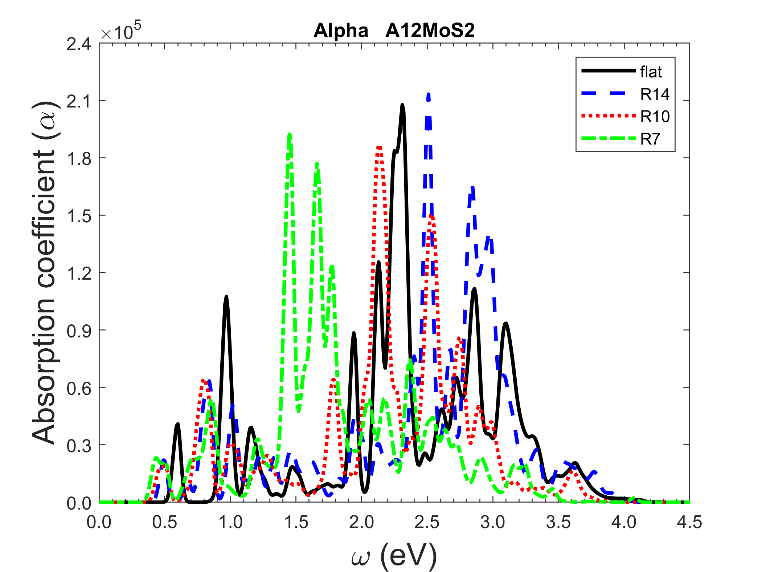


Figure S24. The electron energy loss spectrum (EELS) and absorption coefficient of A12MoS_2_ nanoribbon under different bending curvatures.

The following figure set SA listed below show the band structures of A12MoS2 and A13MoS2 nanoribbons evaluated with PBE and PBE+SOC. The left column is from PBE only and the right column is from PBE+SOC.

Figure set SA


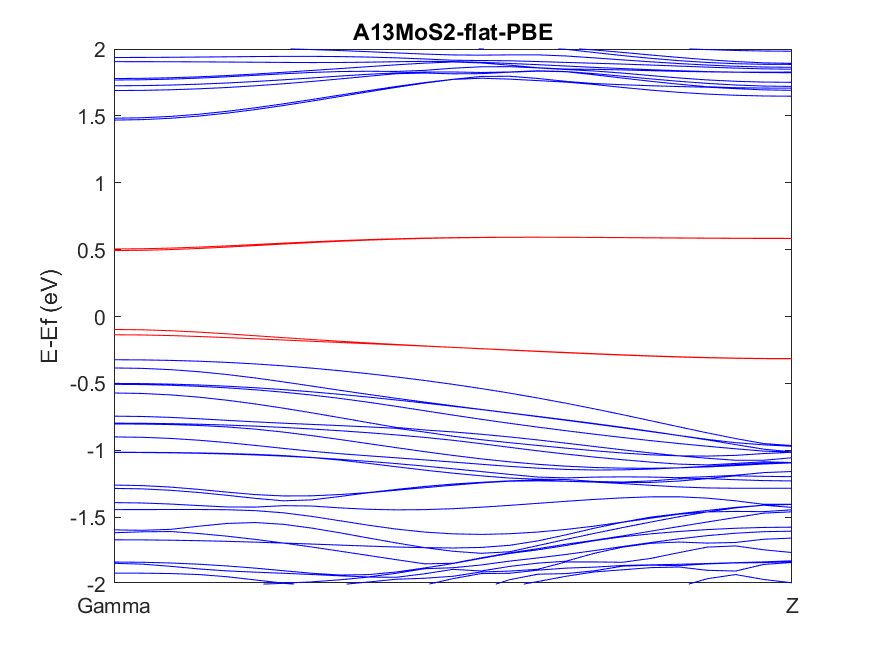

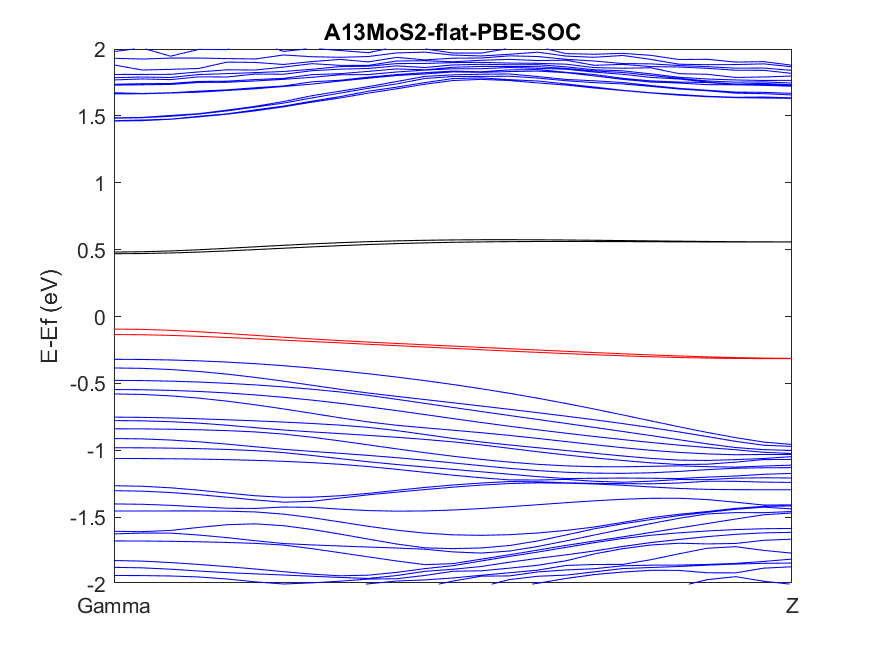


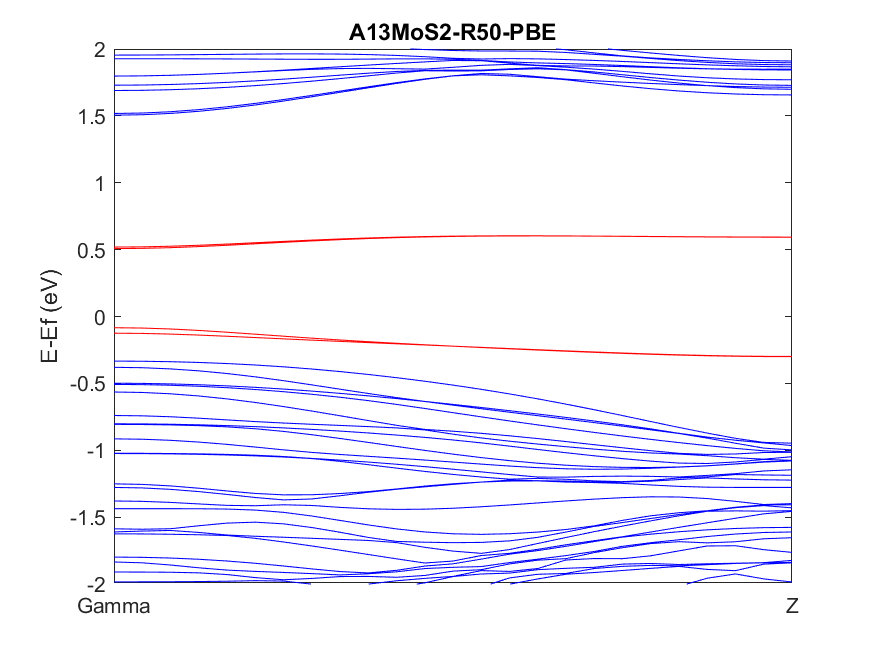

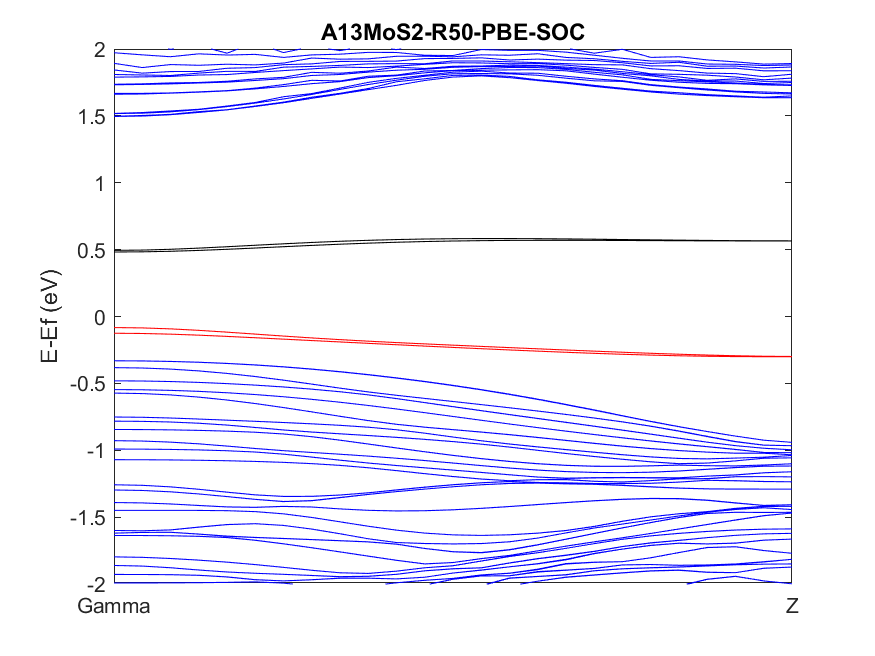


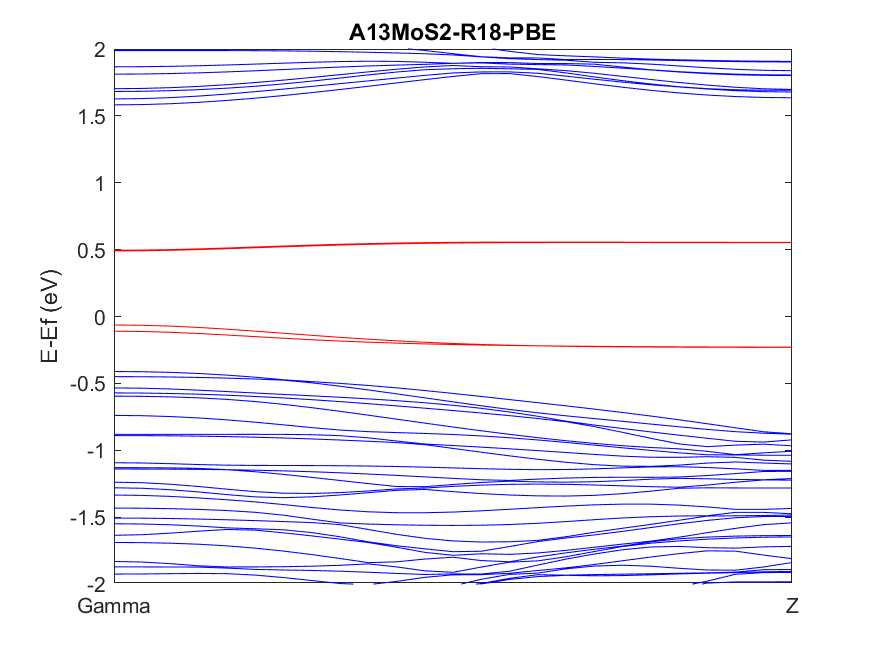

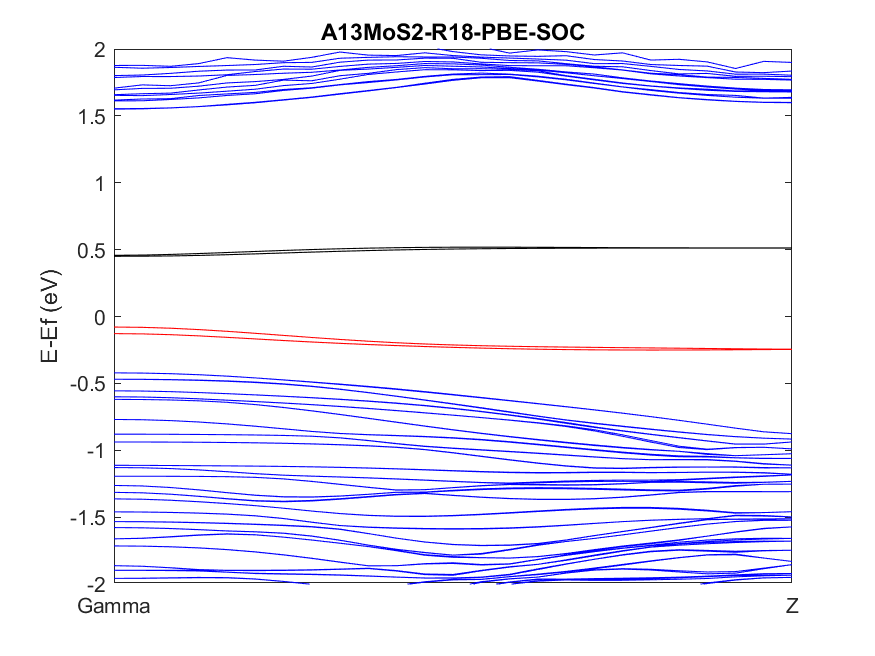


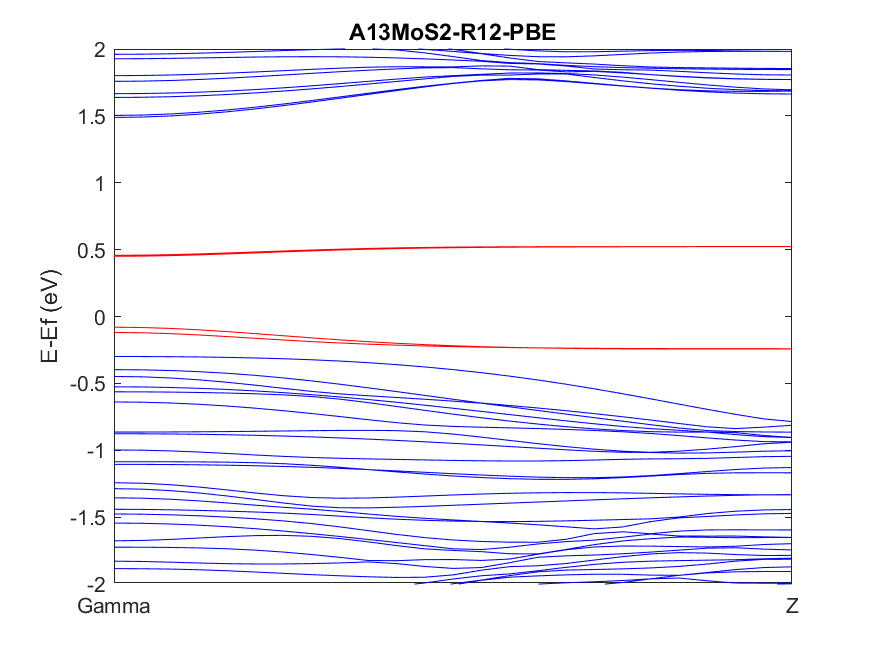

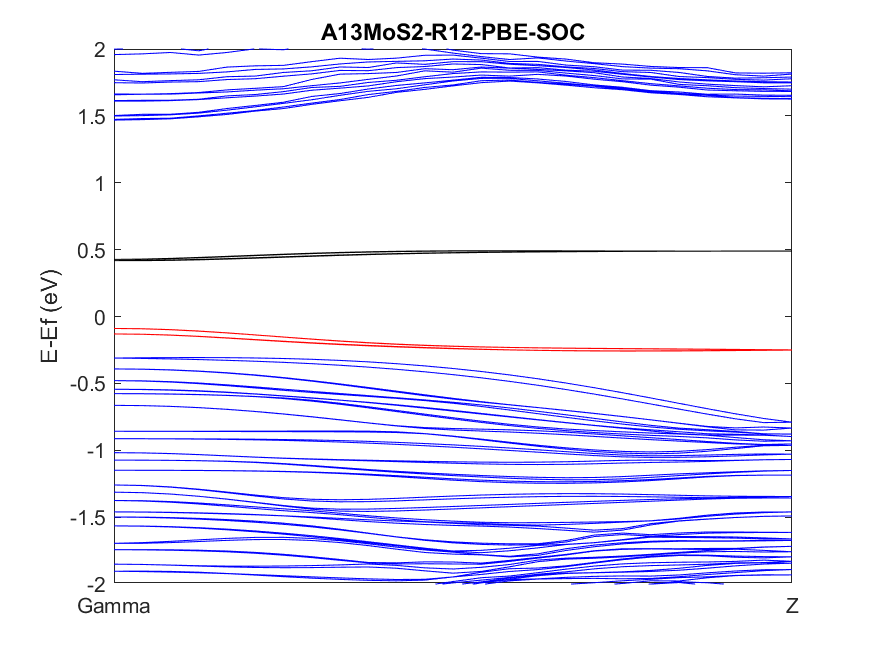


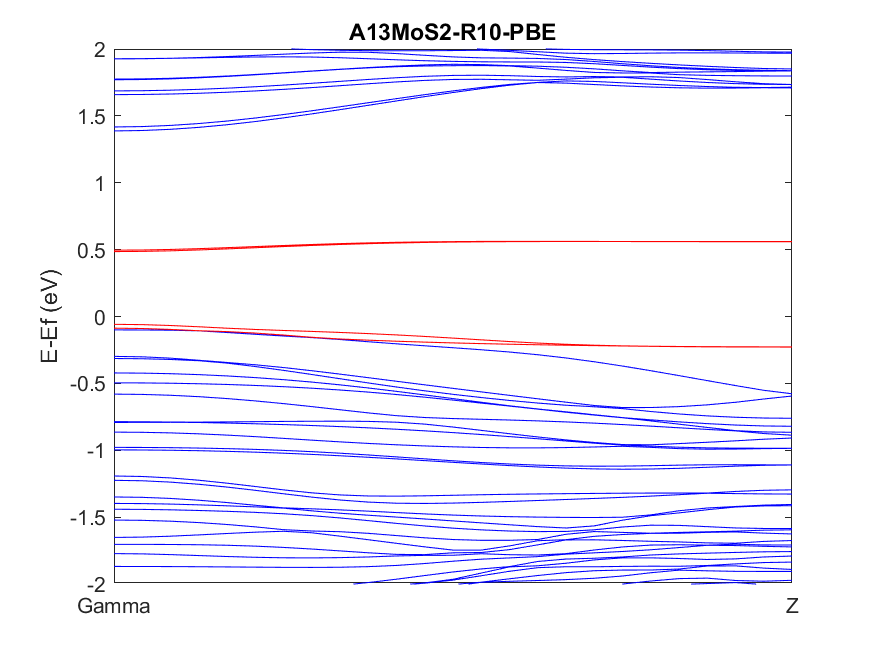

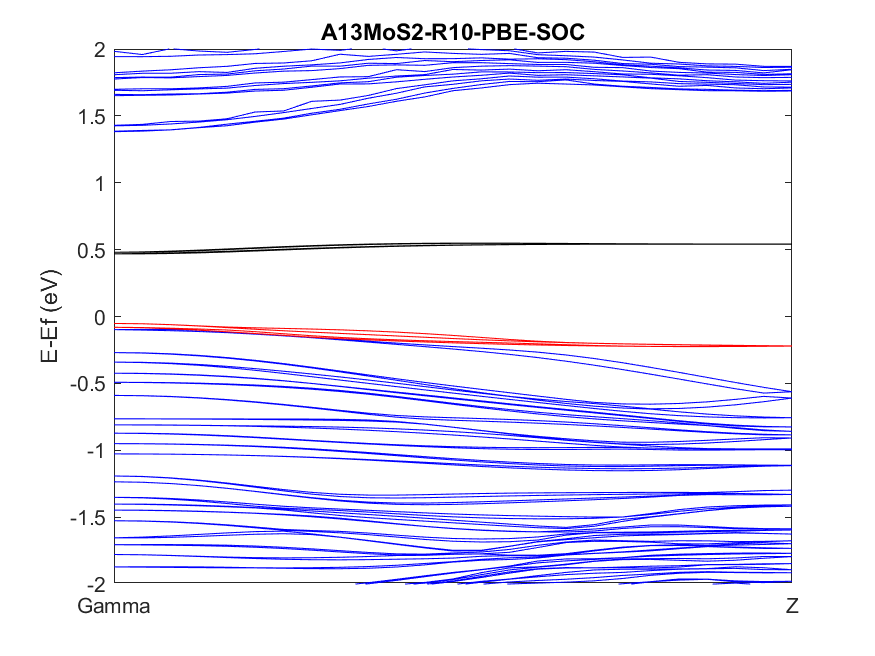


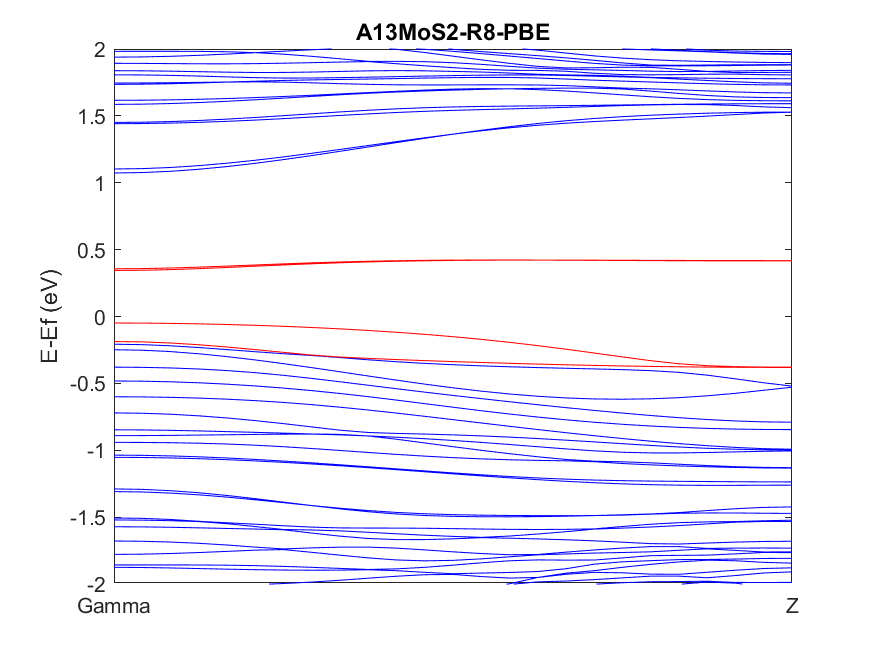

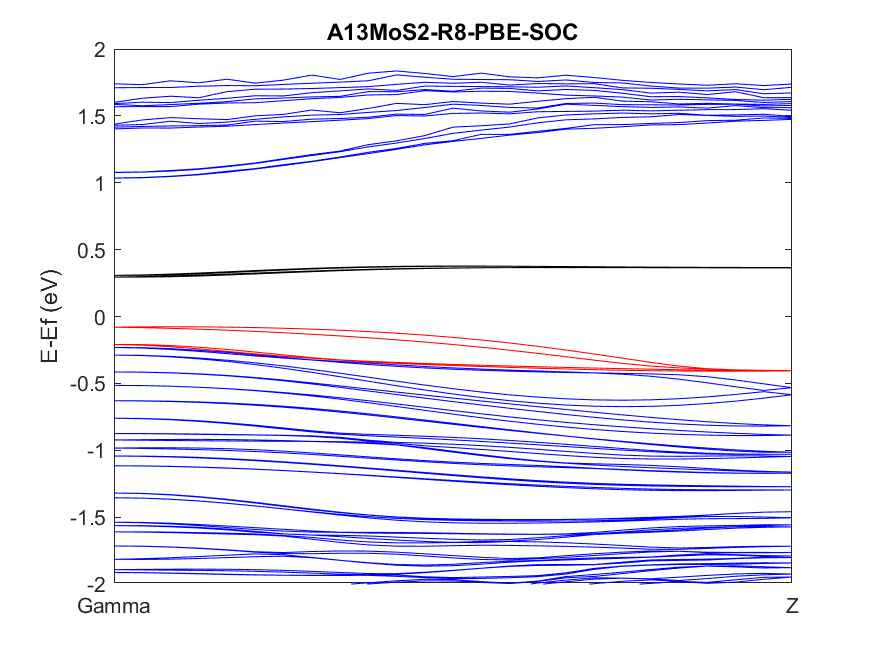


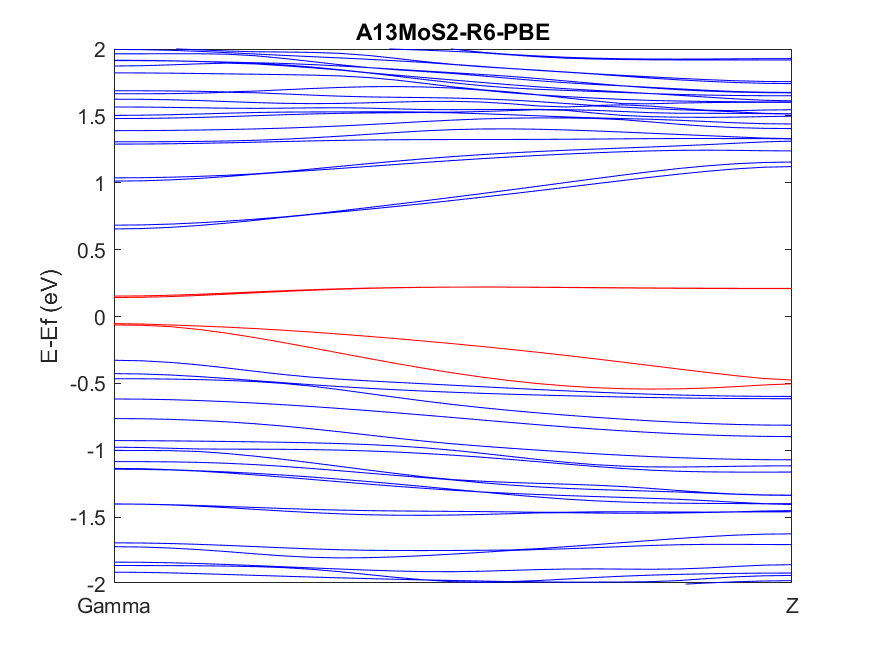

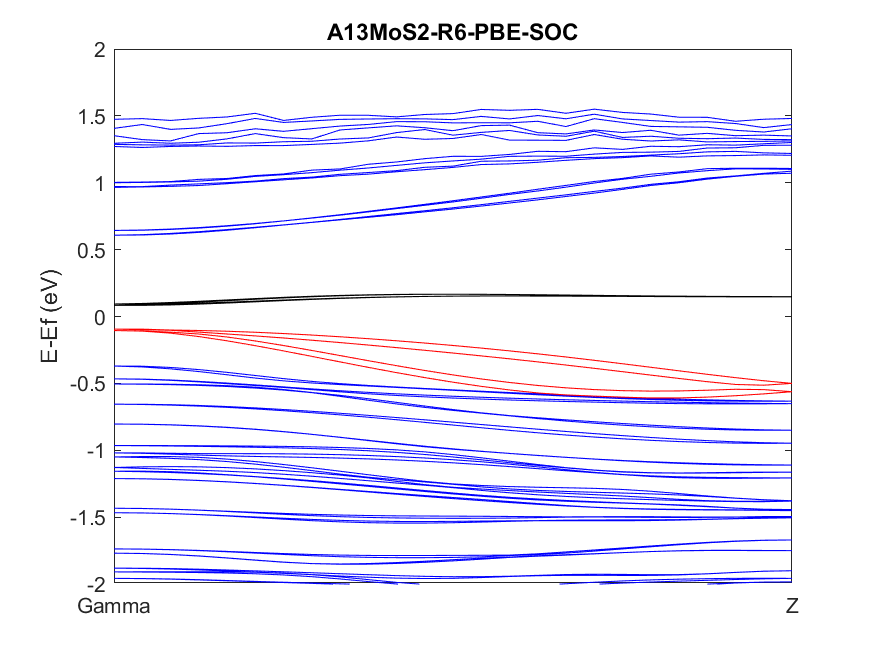


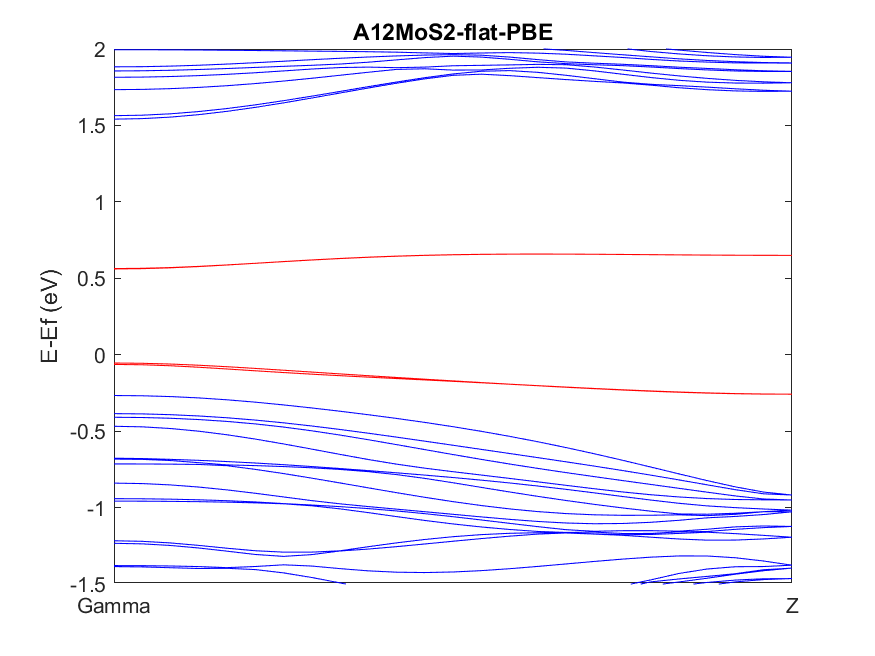

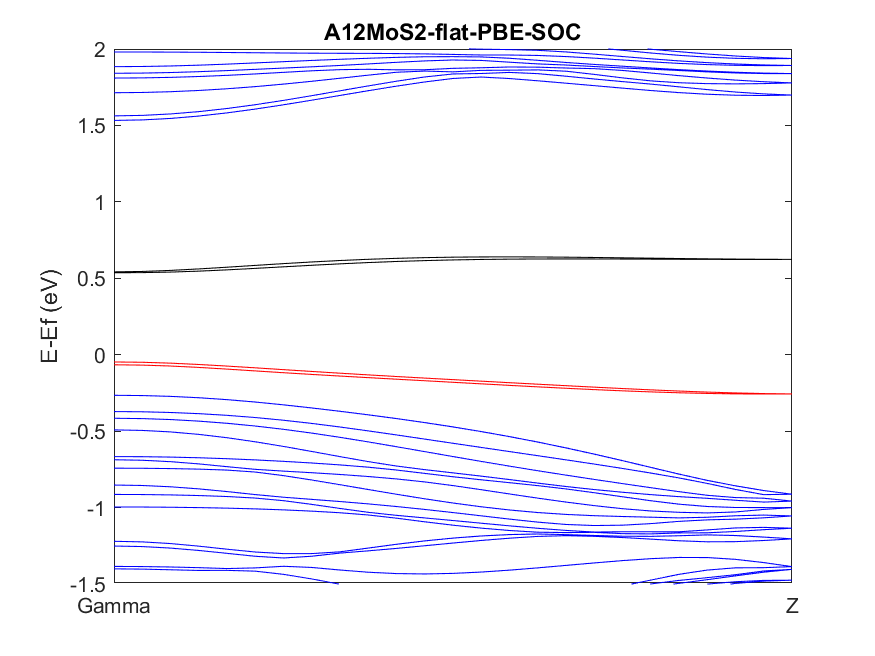


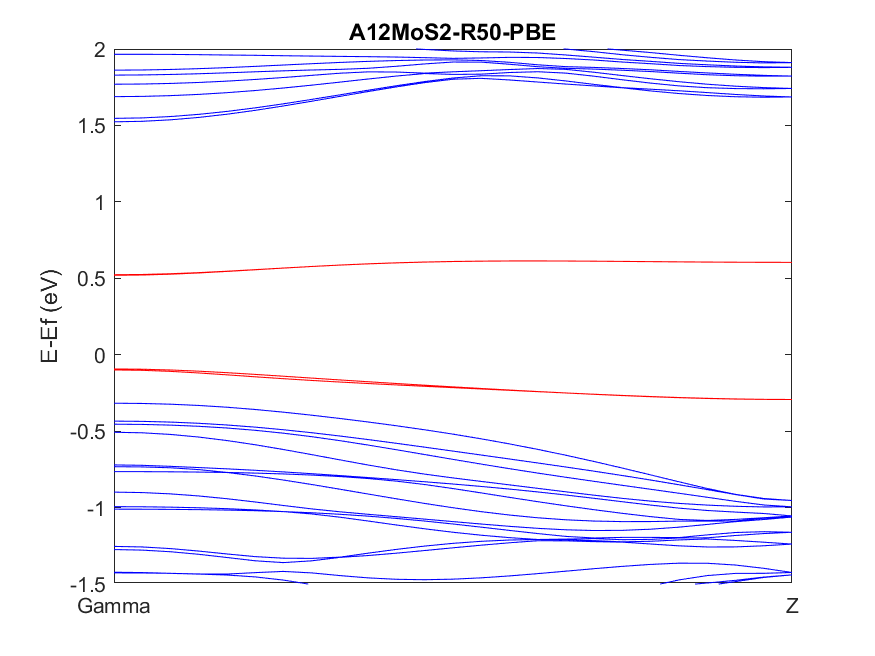

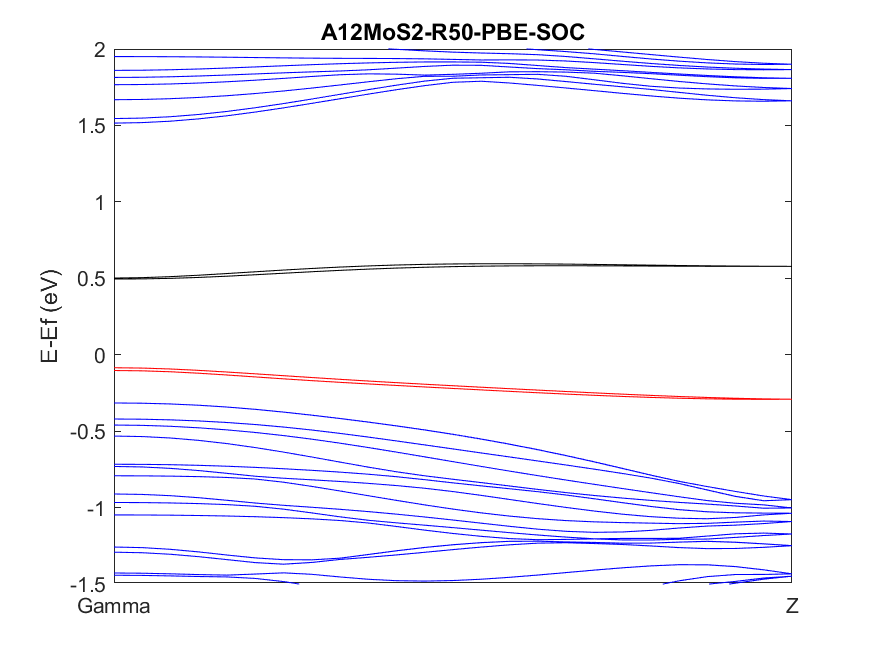


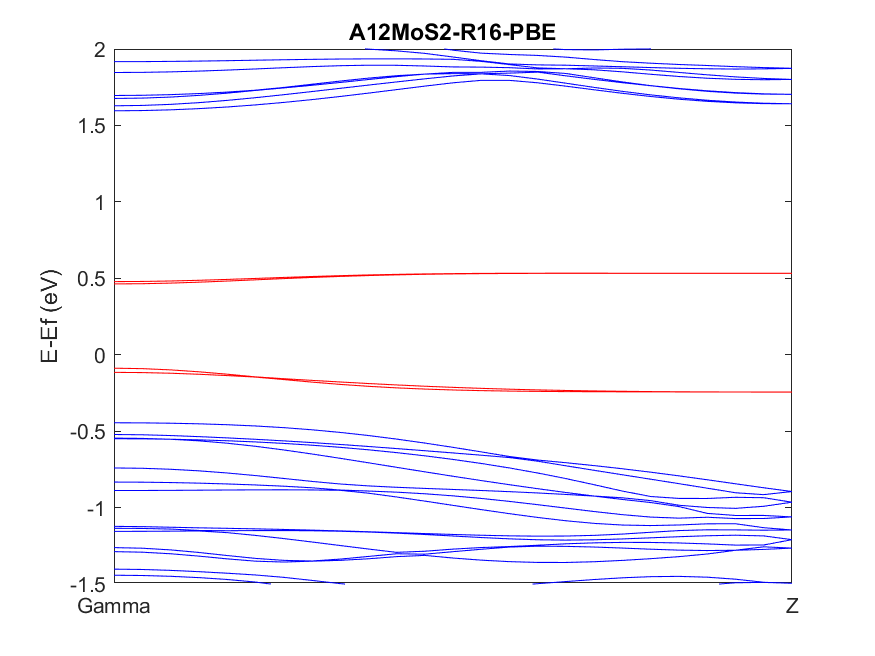

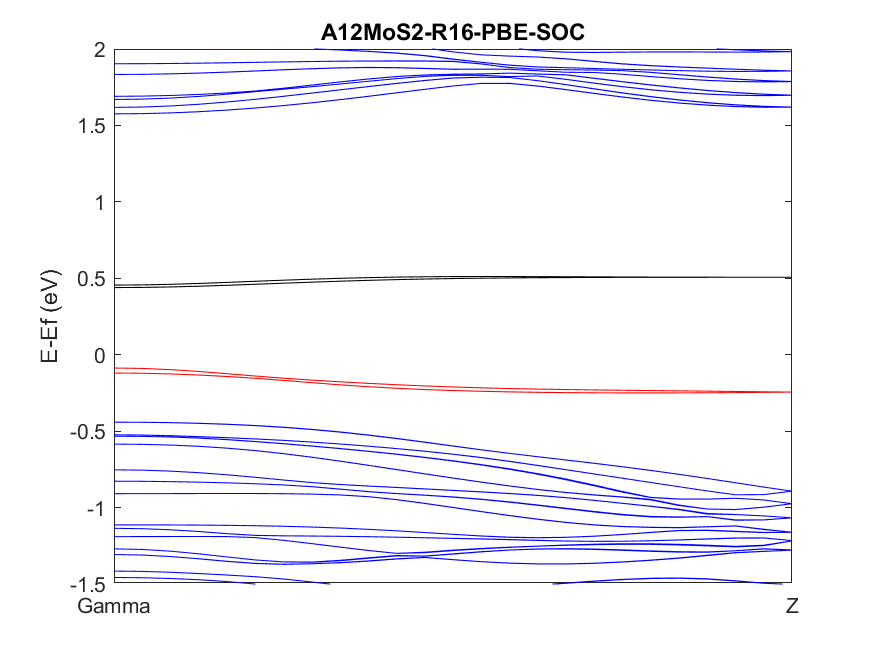


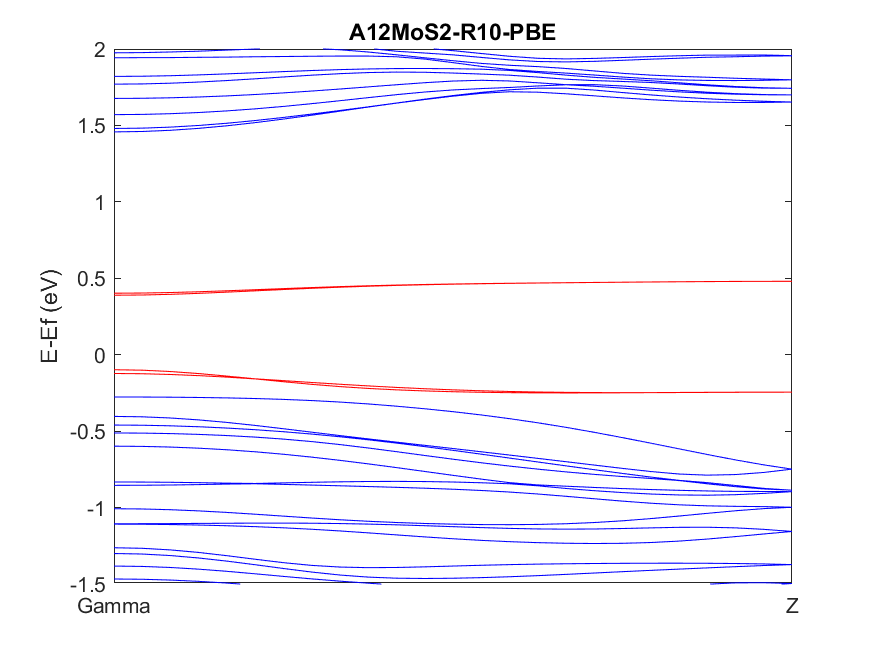

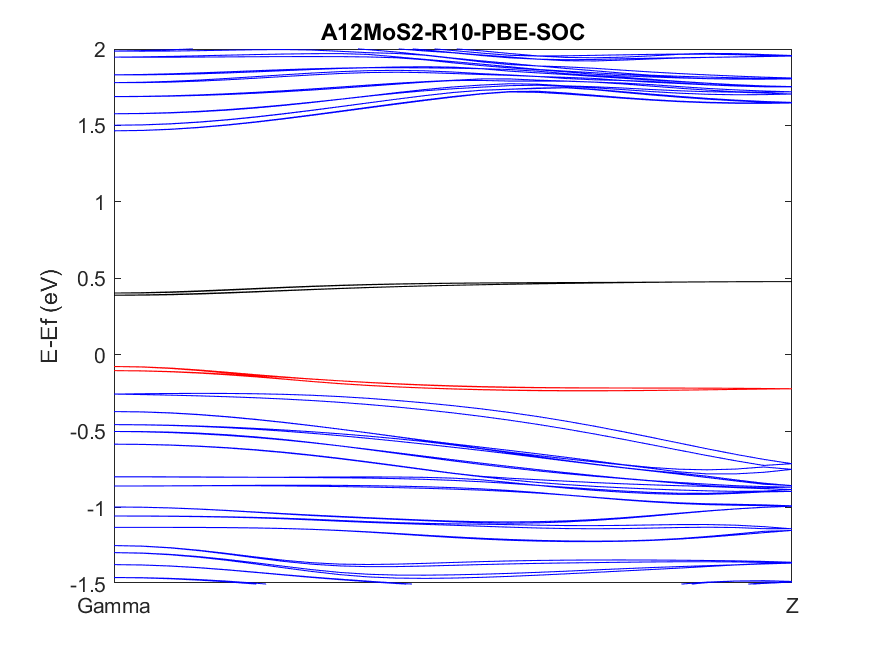


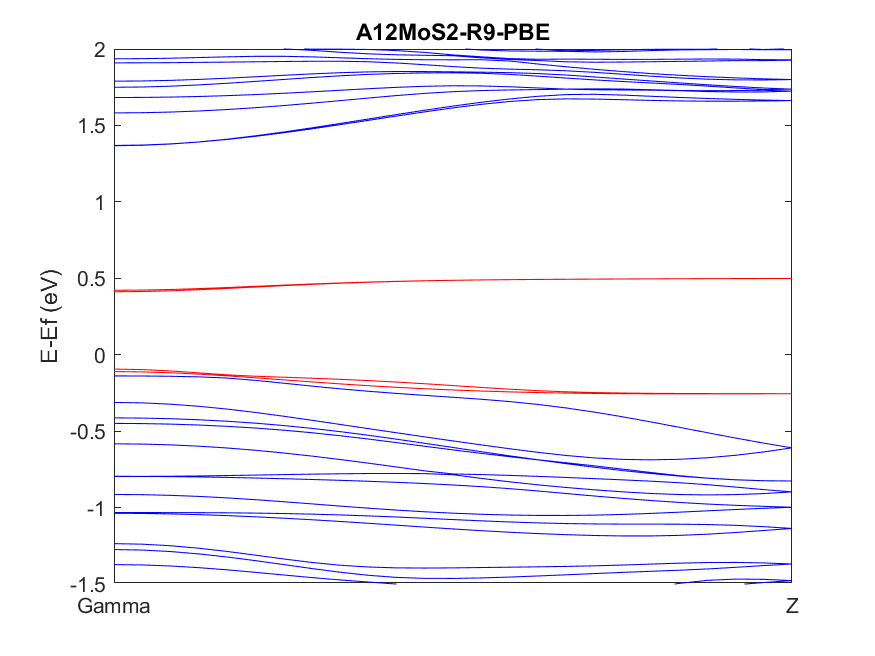

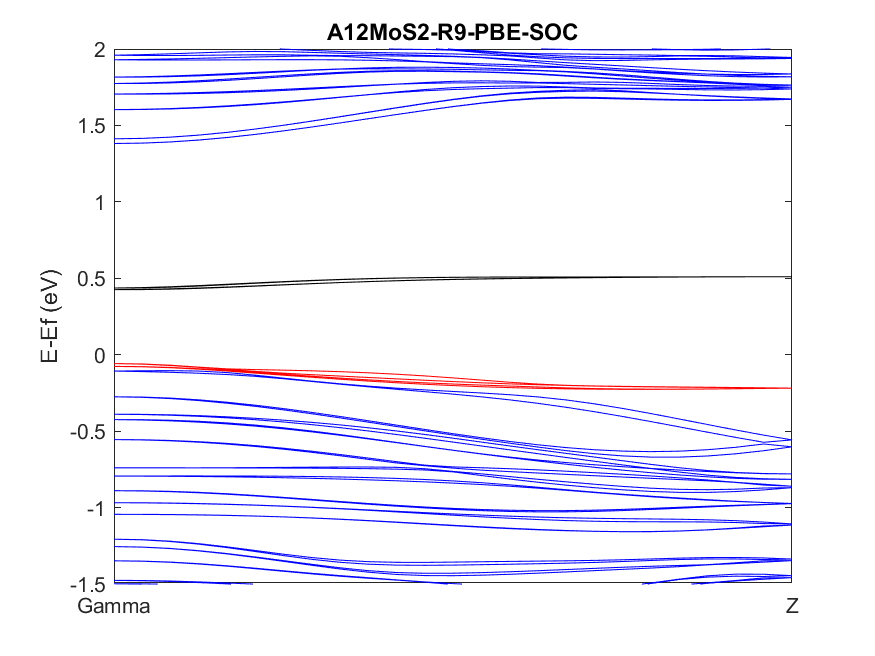


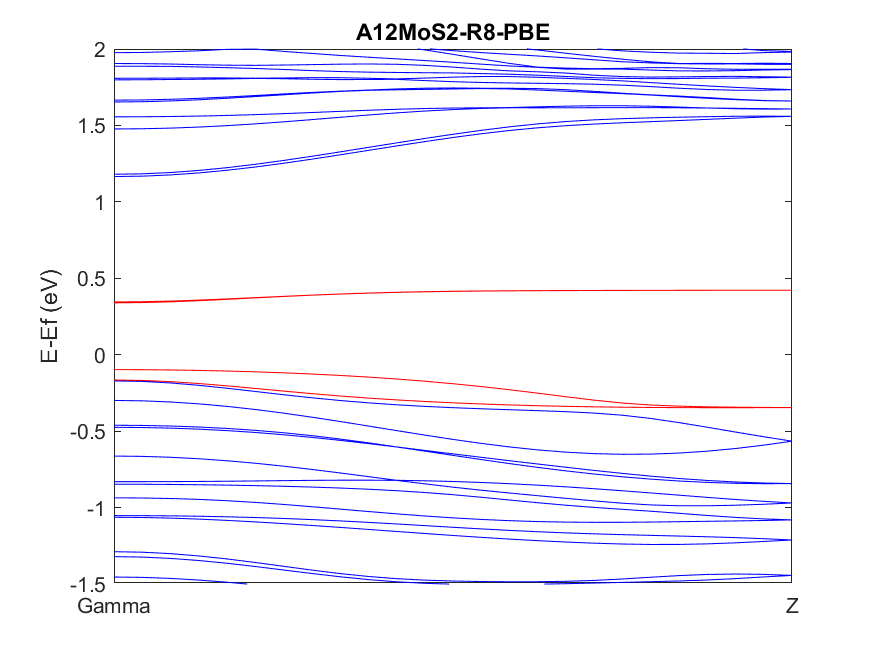

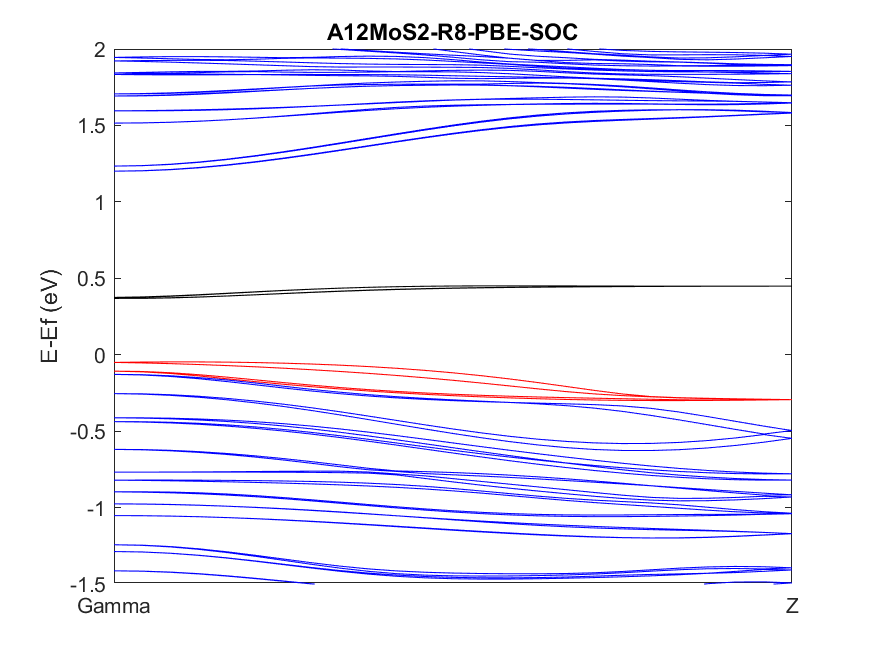


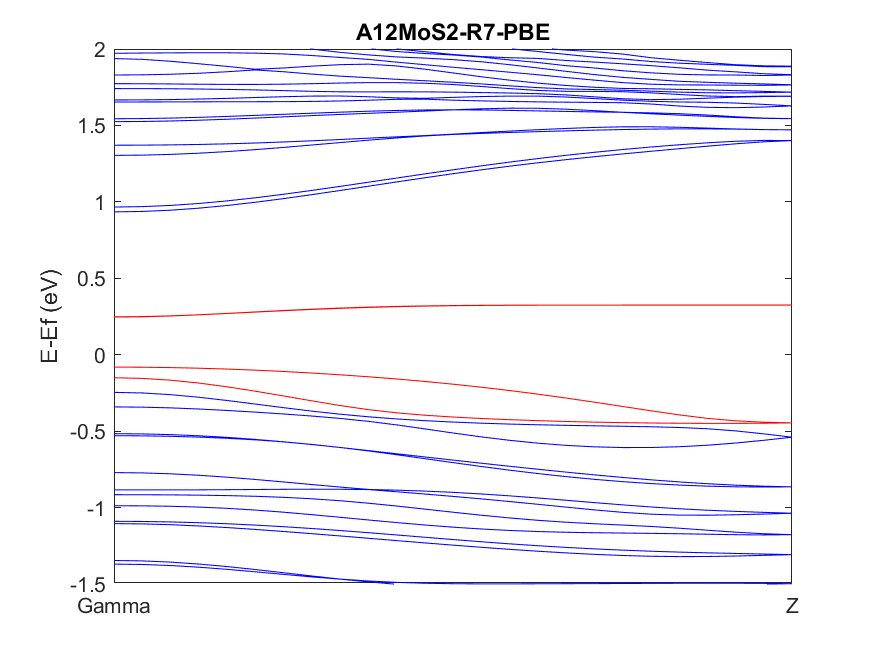

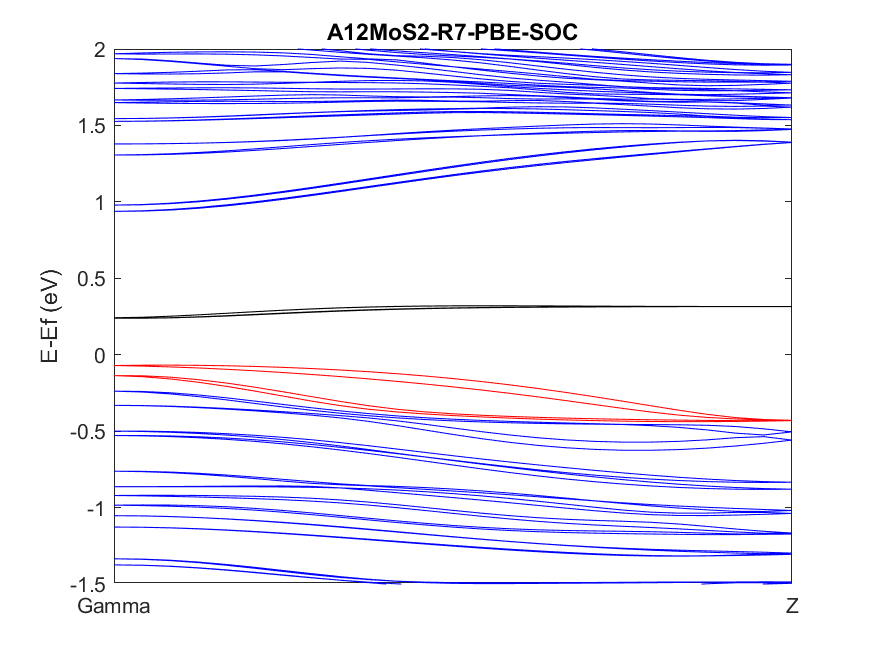

Supplement: Supplementary file 1 — Supplementary Figures. [file 41598_2022_6741_MOESM1_ESM.docx]
